# 3D and 4D assembly of functional structures using shape-morphing materials for biological applications

**DOI:** 10.3389/fbioe.2024.1347666

**Published:** 2024-03-28

**Authors:** Soheyl Mirzababaei, Lily Alyssa Kera Towery, Molly Kozminsky

**Affiliations:** ^1^ Department of Chemical and Biological Engineering, Iowa State University, Ames, IA, United States; ^2^ Nanovaccine Institute, Iowa State University, Ames, IA, United States

**Keywords:** shape-morphing materials, smart materials, origami, kirigami, minimally invasive surgery, biosensors, tissue engineering

## Abstract

3D structures are crucial to biological function in the human body, driving interest in their *in vitro* fabrication. Advances in shape-morphing materials allow the assembly of 3D functional materials with the ability to modulate the architecture, flexibility, functionality, and other properties of the final product that suit the desired application. The principles of these techniques correspond to the principles of origami and kirigami, which enable the transformation of planar materials into 3D structures by folding, cutting, and twisting the 2D structure. In these approaches, materials responding to a certain stimulus will be used to manufacture a preliminary structure. Upon applying the stimuli, the architecture changes, which could be considered the fourth dimension in the manufacturing process. Here, we briefly summarize manufacturing techniques, such as lithography and 3D printing, that can be used in fabricating complex structures based on the aforementioned principles. We then discuss the common architectures that have been developed using these methods, which include but are not limited to gripping, rolling, and folding structures. Then, we describe the biomedical applications of these structures, such as sensors, scaffolds, and minimally invasive medical devices. Finally, we discuss challenges and future directions in using shape-morphing materials to develop biomimetic and bioinspired designs.

## 1 Introduction

With advances in tissue and biomedical engineering, there is a growing demand for functional structures. Several methods, such as electrospinning (Adadi et al., 2020), microfluidics (Navaei-Nigjeh et al., 2023), and (bio)printing (Kirillova et al., 2022), have been used to fabricate structures for tissue scaffolds, temporary or permanent implants, sensors, and minimally invasive devices. While improvements in some properties of these structures, such as biocompatibility and mechanical properties, fabricating sensors, scaffolds, and tissues that meet the needs of complex biological can laborious and methodologically difficult.

One approach to overcome this problem is the application of shape-morphing materials. These materials can respond to a certain stimulus, such as pH, temperature, or magnetic field, which enables change in the structure of the final product. Concepts that have been used to develop functional structures based on these materials include origami and kirigami, Japanese techniques for creating 3D structures from 2D substrates such as paper. While origami is based on folding, kirigami takes advantage of cuts to increase the degrees of freedom and create more intricate structures from planar objects. By designing strategic cuts and folding segments in 2D objects made of shape-changing materials, it is possible to apply a stimulus that results in 3D architecture.

Developing 3D assemblies based on shape-morphing materials has received attention in fabricating structures with biological applications since it enables analogous structures to tissues and organs in a relatively straightforward fashion. Additionally, the shape-changing property has been used for other applications, such as a measurable signal for (bio)sensors (Yamagishi et al., 2019) or devices that reduce the invasiveness of surgeries (Breger et al., 2015). Accordingly, in this article, we first describe two frequently-used methods to develop such structures, lithography and bioprinting. Then, we discuss common structures that have been developed based on these methods. We summarize several important biological applications of these structures and how they can address existing problems in their respective fields.

## 2 Manufacturing methods

Kirigami- or origami-inspired techniques are often applied to a two- or three-dimensional precursor on an underlying sacrificial layer, facilitating subsequent release from a planar supporting substrate. 4D multiscale structures can be fabricated using approaches that range from macroscale (e.g., manual assembly, molding, printing, and deploying-stacking) to nanoscale (e.g., photolithography, laser writing, nanoimprinting, and bottom-up synthesis). Due to their role as the foundation of many shape-morphing structures, we will highlight fabrication strategies that are widely applied across shape-changing technologies: lithography and bioprinting (Table 1).

**TABLE 1:** Comparison of manufacturing methods.

Manufacturing methods		Pros	Cons	Applications	References
Lithography	Photolithography	• Different modes (positive vs. negative photoresist)	• Sample must be able to withstand UV light	• Master mold fabrication	Romeo and Cantù (2005)
		• Design flexibility based on mask	• Challenges in achieving maximum resolution due to photoresist swelling, alignment challenges, debris	• Microlenses	O’Brien et al. (2001)
		• High throughput		• Cell culture platforms	O’Brien et al. (2001)
		• Well-established and widely available			Thompson et al. (1983)
					Del Barrio and Sánchez-Somolinos (2019)
	Electron beam (E-beam)	• High resolution (order of nanometers)	• Requires specialized equipment	• Integrated circuits	Thompson et al. (1983)
		• Design flexibility based on mask	• Low throughput	• Photonic crystals	Altissimo (2010)
				• Nanochannels	
	Soft lithography	• Relatively inexpensive	• Requires master mold	• Pattern stamping	Qin et al. (2010)
		• Accessible	• Lower resolution		
Bioprinting	Extrusion	• 3D structures	• Limited to high viscosity “inks”	• Cell bioprinting	Rider et al. (2018)
		• Rapid generation of high cell density patterns	• Limited resolution	• Restoration of ovine calvarial defects	Chien et al. (2013)
			• Cell exposure to shear stress		
	Inkjet	• Relatively high spatial resolution	• Limited to high viscosity “inks”	• Cartilage formation	Xu et al. (2012)
			• Cell aggregation within bioink	• Scaffolds	Cui et al. (2012a)
				• *In-situ* printing	Ku, (1997)
					Cui et al. (2012b)
					Rider et al. (2018)
	Large-area-laser-based vapor deposition, e.g., matrix-assisted pulsed-laser evaporation direct-write (MAPLE DW)	• 3D structures	• Negative effects of pressure and shear on cells	• Cell and structure bioprinting	Chrisey et al. (2000)
		• High printing resolution	• Throughput		Yang et al. (2021)
					Li et al. (2016)

### 2.1 Lithography

**FIGURE 1:**
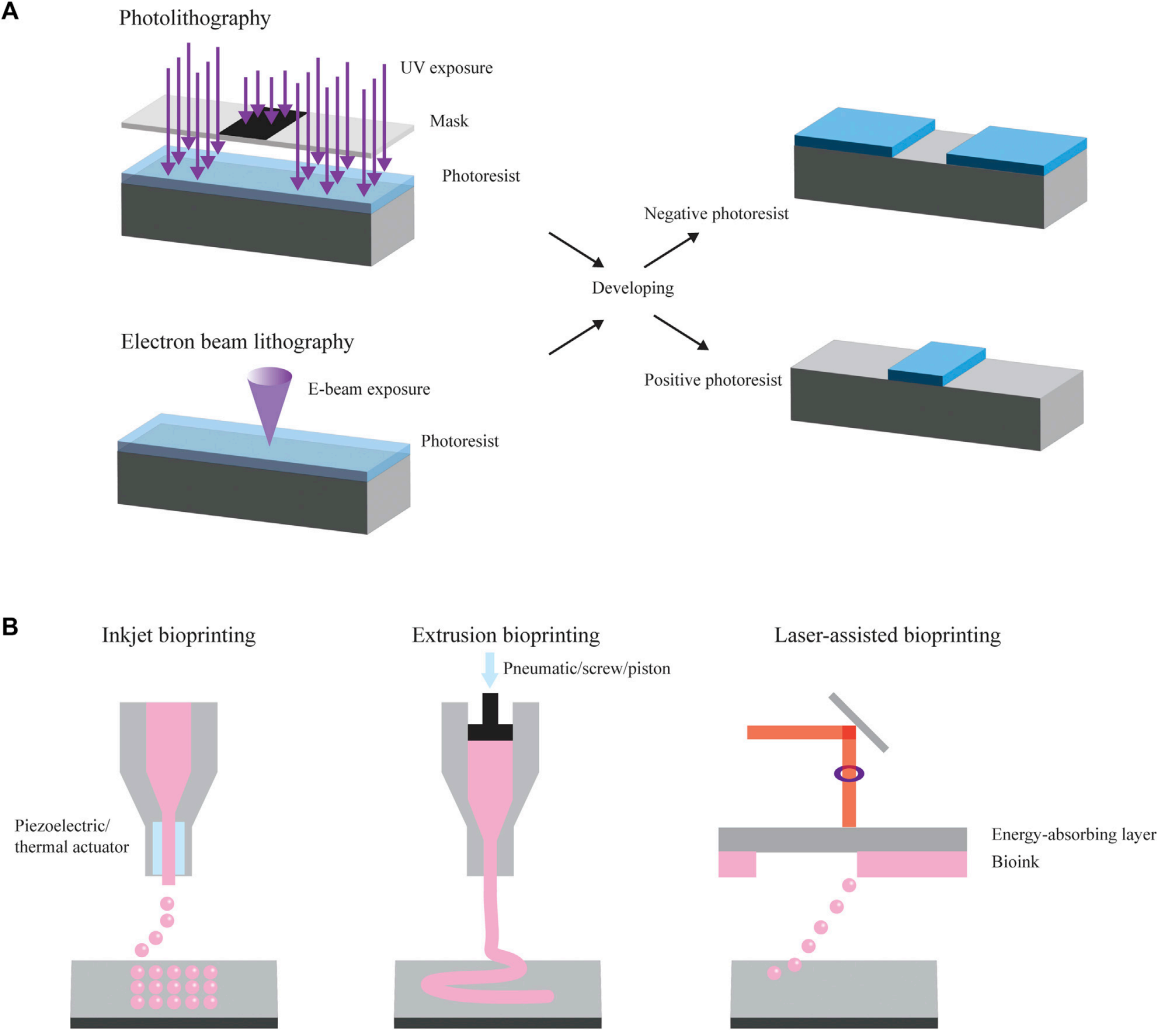
Manufacturing methods. **(A)** Photolithography and electron beam (E-beam) lithography are common methods used to create and modify substrates that can ultimately be used in shape-changing applications. They make use of selective patterning using a mask. They also often form the basis for components of soft lithography (not pictured). **(B)** Common examples of bioprinting include inkjet bioprinting, extrusion bioprinting, and laser-assisted bioprinting.

Lithography is a fundamental technique used in micro- and nanofabrication to create intricate patterns and structures on various substrates. Different types of lithography include photolithography, electron beam (E-beam) lithography, and X-ray lithography (Thompson et al., 1983; Altissimo, 2010; Qin et al., 2010) (Figure 1A). In different shape-morphing applications, they have served both to define the shapes of two-dimensional substrates and to expose sacrificial layers.

One of the most common forms of lithography is photolithography, a technique applied to transfer patterns from a mask onto a two-dimensional substrate using light. To start, a light-sensitive material known as photoresist is spin-coated onto a substrate, such as a silicon wafer or a glass slide. Following this spin coating process, the substrate undergoes a heating process, referred to as soft baking, which partially evaporates the solvent and stabilizes the photoresist. Subsequently, the sample is exposed to UV light of a wavelength ranging from 150 to 450 nm (depending on the photoresist) using a mask featuring the desired pattern, leading to the selective patterning of the photoresist (Romeo and Cantù, 2005). When the sample is exposed to UV light, the exposed photoresist either becomes soluble or insoluble in photoresist developer due to a radiation-induced solubility change. A positive photoresist (e.g., AZ 4562) describes a material where exposure to light renders it soluble in the developer, while the unexposed section remains insoluble. Conversely, a negative photoresist, which is commonly used in polydimethylsiloxane (PDMS) replica molding, results in the region exposed to light becoming insoluble in the developer and the unexposed portion dissolving when treated with the developer (O’Brien et al., 2001). The final step is the development process, where the sample is immersed in a solution to remove only the soluble photoresist. This process can be repeated to create subsequent layers with different patterns. Photolithography can provide high throughput and high resolution with high accuracy in transferring a mask pattern onto the photoresist (Thompson et al., 1983). However, the maximum resolution capability is seldom achieved because of photoresist swelling, difficulty in alignment, and debris between the mask and wafer. These imperfections can cause undesirable effects reproduced in each subsequent exposure in multilayer fabrication, decreasing the ultimate device throughput and resolution (Thompson et al., 1983). Various applications of photolithography include generating masters molds for reproducing replicate parts, manufacturing microfluidic chips, preparing micro lenses, generating platforms for cell culture studies, and forming microstructures with topographic cues able to guide cell morphology, function, and fate (Del Barrio and Sánchez-Somolinos, 2019).

Higher resolution can be achieved through E-beam lithography, a method of lithography involving a finely concentrated stream of electrons that can be precisely directed across a surface coated with a radiation-sensitive polymer. Analogous to the light in photolithography, a focused electron beam is scanned across a substrate covered in an electron-sensitive material, altering the electron-beam resist solubility characteristics based on the energy deposited by the beam (Thompson et al., 1983). Depending on whether the resist is positive or negative, areas that are exposed or unexposed, respectively, are removed during development. When the electron beam enters the substrate, it loses energy due to elastic and inelastic collisions, a phenomenon called electron scattering. These scattering processes lead to the beam’s broadening and result in the resist being exposed at points far from the initial electron interaction, causing the resulting patterns to be wider than anticipated. Various exposure models, such as the Gaussian beam and Shaped beam, have been developed to predict resist profiles and enhance precision. E-beam lithography offers resolution on the order of nanometers, typically operating with currents ranging from 0.05 to 0.5 picoamperes. These systems can be categorized into two types: those employing scanned, focused electron beams that sequentially expose the wafer, and those projecting an entire pattern all at once onto a wafer (Altissimo, 2010). The performance capabilities of each of these subsystems, along with the limitations posed by electron scattering, determine the overall resolution and fidelity of an E-beam lithographic system. Challenges associated with electron scattering often result in reduced resolution or throughput. However, current proprietary software can convert pattern data into a machine-readable file containing all the necessary instructions for directing and scanning the beam, ensuring accurate exposure and increased precision. E-beam lithography applies to the fabrication of integrated circuit production, photonic crystals, and channels for nanofluidic experiments (Thompson et al., 1983; Altissimo, 2010).

Soft lithography builds upon these other lithographic techniques. In soft lithography, a pattern is developed into a mask and master mold, allowing the subsequent fabrication of a PDMS stamp to be used in the generation of micro- and nanostructures using the stamp via methods like printing, molding, and embossing. The initial pattern can be represented through computer-aided design (CAD) software applications like Autodesk AutoCAD and DesignCAD and then printed as a mask using a high-resolution printer. Typically, a commercial printer with high resolution can produce lines as thin as 20 µm with acceptable edge quality, providing a convenient means to create photomasks on transparent films (Qin et al., 2010). When even higher resolutions, such as 1 µm or submicron, are required, chrome photomasks may be required. Master fabrication necessitates techniques like photolithography or electron-beam lithography to create a master featuring patterned relief structures on its surface. The creation of the PDMS stamp involves pouring a liquid precursor over a master surface with complementary structures. In principle, any elastomer could be used, but the majority of work focuses on cross-linked PDMS. Composite stamps consist of two layers—a rigid layer (typically 30- to 40-µm-thick PDMS) supported by a flexible layer (3- to 5-mm-thick Sylgard 184 PDMS) (Qin et al., 2010). While methods like photolithography and electron-beam lithography are often costly and require frequent access to clean room facilities, soft lithography offers a more straightforward and accessible approach, enabling experiments to be conducted in a standard chemical laboratory setting, aside from the master fabrication process.

### 2.2 Bioprinting

Bioprinting is another advanced technology merging the principles of three-dimensional (3D) printing with the field of biology to create 3D living structures. The general process of bioprinting entails the precise deposition of living cells, biomaterials, and growth factors layer by layer to construct functional tissues, organs, or other biological constructs. Different types of bioprinting include inkjet-, and laser-, and extrusion-based bioprinting (Figure 1B).

Extrusion-based bioprinting stands as the most widely employed printing method. Typically, it involves the extrusion of ink through a printhead to construct a 3D structure in a layer-by-layer fashion. This process occurs through the use of a piston, screw, or pneumatic pressure mechanism. Highly viscous bioinks—biological cells suspended in hydrogels such as PDMS and polycaprolactone (PCL) (Rider et al., 2018)—can be printed through micro-nozzle sizes. The precise control over cell deposition, distribution rate, and production speed has significantly expanded the utility of this technology for scaffold production. Its primary advantage lies in the capacity to achieve high cell densities rapidly. The continuous deposition of bioink ensures robust structural integrity. However, this method is limited to materials with high viscosity to maintain filamentous structure after deposition. Some adjustments, including material concentration, nozzle pressure, and diameter, can partly mitigate this limitation. In extrusion-based bioprinting, cells are exposed to shear stress when passing through the nozzle and pressure while in the syringe prior to extrusion. Nonetheless, the resolution of extrusion-based printing is approximately 200 μm, which is notably lower than that of inkjet- and laser-based bioprinting (Rider et al., 2018). One application of extrusion-based technology for generating specific 3D patterns is pressure-assisted bioprinting (PAB). PAB has been employed in the bioprinting of cells and organs, demonstrating the preserved activity of printed materials. The range of bioprinted cells encompasses mouse pre-osteoblasts, human mesenchymal stem cells (MSCs), endothelial cells (ECs), and osteogenic progenitors. These bioprinted cells have been effectively utilized in the restoration of ovine calvarial defects (Chien et al., 2013).

Inkjet-based bioprinting is a non-contact printing technique in which droplets of dilute solutions are dispensed, driven by thermal, piezoelectric, or microvalve processes. This technology is based on the conventional inkjet process used by desktop inkjet printers, whereby individual droplets are used to pattern a substrate. A structure is formed by continuously depositing droplets at predesigned points, enabling a structure with irregular shapes to be easily fabricated. High spatial resolution can be achieved between 50 and 300 μm; however, cell aggregation within the bioink can change droplet formation and trajectory, thereby reducing printing quality. Similar to extrusion-based bioprinting, this method is limited to high viscous materials to maintain filamentous structure. Applications of inkjet-based bioprinting have successfully demonstrated cartilage formation in mice (Xu et al., 2012), FGF-2 and FGF-2/TGF-β1 doped scaffolds for cartilage development (Cui et al., 2012a), all-in-one solution for printing skin (Ku, 1997), and *in situ* printing to repair full thickness skin wounds on backs of mice (Cui et al., 2012b); applications that do not make use of shape transformation are reviewed elsewhere (Rider et al., 2018).

Finally, an additional commonly used bioprinting approach employs large-area-laser-based vapor deposition technology. One example of this printing technique, the matrix-assisted pulsed-laser evaporation direct-write (MAPLE DW), deposits thin and uniform polymer films without significant decomposition (Chrisey et al., 2000). The process of cell deposition through MAPLE DW can be divided into three stages: droplet formation, travel, and landing. In the droplet formation stage, induced bubble collapse is used to create a shockwave, projecting a droplet off the print ribbon. This step can generate substantial pressure, leading to a stress wave as the bubble is confined in rigid media, potentially causing damage to the cell membrane. Following this, the droplet enters the travel or free-fall phase. Ultimately, the collision of the droplet onto a hydrogel-coated receiving surface involves two impacts: the droplet collides with the substrate, and the cells within the droplet collide with the substrate. This MAPLE DW process can produce high-quality, high-resolution (±5 µm) polymeric, organic, and biomaterial films on various types of substrates. One noteworthy limitation of the initial iteration of this method is the detrimental effect of shear stress on the cells during deposition, leading to lower-than-desirable viability. However, adjustments to parameters, such as laser settings, have resulted in consistent cell viability exceeding 90%. Another notable drawback of this method is the printing speed for structures and the constraints on the size of the constructs that can be produced (Yang et al., 2021). Employing laser-based bioprinting, scientists have showcased their capability to 3D print various cells, including human dermal fibroblasts, mouse C2C12 myoblasts, bovine pulmonary artery ECs, breast cancer (MCF-7) cells, and rat neural stem cells (Li et al., 2016).

Bioprinting holds great promise in sensing and *in vitro* models, though based on the application, there are several design parameters that must be considered. Material biocompatibility is crucial in applications that require implantation or the maintenance of living cells. A primary limitation of bioprinting is the lack of bioinks that are suitable for bioprinting. Hydrogels are the primary biomaterial used for bioink printing; they may be either natural (e.g., gelatin, alginate, agarose, chitosan, dextran, fibrinogen, hyaluronic acid, etc.) or synthetic [e.g., poly(ethylene glycol)s (PEGs), Pluronics, polyacrylamide, poly(2-hydroxyethyl methacrylate) (PHEMA)]. While natural hydrogels have improved bioactivity, synthetic hydrogels are cheaper and easier to modify (Gillispie et al., 2020). The properties of these materials can then be used to build towards different transformations of interest. Beyond the desired mechanochemical properties for the structure, cell viability during and after printing is important. Highly viscous bioink could impose higher shear stress on cells in inkjet- and extrusion-based printing. The cell-laden bioink will flow through low-diameter cylindrical geometries, an additional source of shear stress. In laser-assisted printing, the shear stress results from the formation of the jet and landing of the bioink on the substrate. Additionally, thermal and radiation treatments for crosslinking could negatively impact cell viability. More details about the effect of different bioprinting methods on cell viability are discussed in other review articles (Hull et al., 2022; Xu et al., 2022).

## 3 Common structures

**FIGURE 2:**
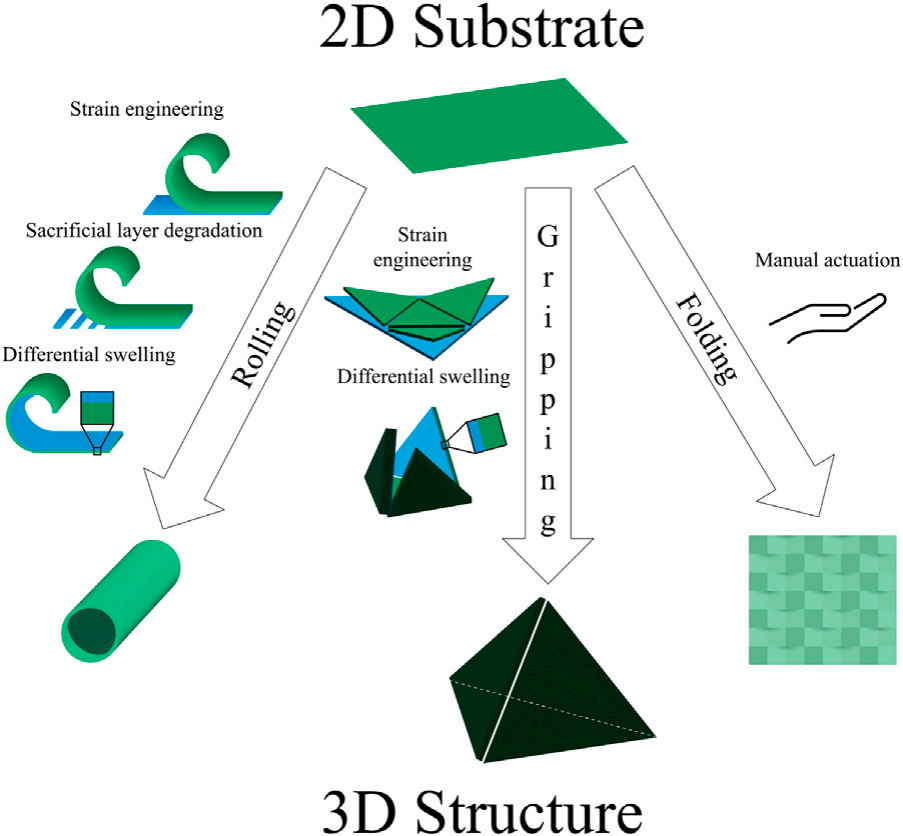
Common structures. The toolbox of shape-morphing strategies contains several techniques used to induce rolling, gripping, and folding. Examples of actuation include strain engineering, sacrificial layer degradation, differential swelling, and manual folding.

In converting from a two-dimensional substrate to a three-dimensional structure, researchers make use of a variety of preliminary shapes and strategic transformations. Due to frequently used actuation principles and applications, we see certain themes among the resulting shapes, which we have categorized by their transformations: rolling, gripping, and folding (Figure 2; Table 2).

**TABLE 2:** Summary of common transformation strategies to develop functional structures.

Types of structure	Actuation system			Application	References
	Stimuli	Sacrificial layer	Driving force		
Rolling	Dissolving of sacrificial layer	Alginate	π-π interaction	*In vitro* model	Sakai et al. (2019)
	Temperature	NA^a^	Swelling behavior	Cardiac microtissue transplants	Pedron et al. (2011)
	Hydrolysis	NA	Differential swelling ratios	Tissue scaffold	Zakharchenko et al., (2011)
	Removing stress	NA	Pre-stressed layer	Tubular scaffold	Yuan et al. (2012)
	Etching of sacrificial layer	Si or Ge	Differential strain	Neural conduit	Froeter et al. (2014)
	Etching of sacrificial layer	AR P-3510	Differential strain	Single-cell study in confined tubular structure	(Xi et al., 2014; Koch et al., 2015; Xi et al., 2016)
	Etching of sacrificial layer	A mixture of acrylic acid and hydrated LaCl_3_	Differential strain	Neuronal cuff implants	Karnaushenko et al. (2015)
	Releasing from mold	NA	Cellular contractile force	Tubular scaffold	Sang et al. (2017)
	Immersing in aqueous solution	NA	Differential swelling ratios	*In vitro* model	Kwag et al. (2016)
	Immersing in aqueous solution	NA	Differential swelling ratios	Tubular scaffold	Kirillova et al. (2017)
Gripping	Temperature	NA	Differential swelling ratios	Microgripper	Stoychev et al., (2011)
	Etching	Cu	Prestressed layer	Microgripper	Malachowski et al. (2014)
	Temperature	Cu	Prestressed layer	Microgripper	Jin et al. (2020)
	pH or humidity	NA	Differential swelling ratios	Sensor	Zeng et al., (2023)
Folding	NA	NA	Manual folding	Tissue scaffold	Kim et al. (2015)
	NA	NA	Manual folding	Monitoring bacterial growth	Funes-Huacca et al. (2012)
	NA	NA	Manual folding	*In vitro* model	Camci-Unal et al. (2016)
	NA	NA	Manual folding	*In vitro* model	Rodriguez et al. (2023)

^a^ NA: not applicable.

### 3.1 Rolling

In addition to an extrinsic change from 2D to 3D, such as by applying magnetic stimulation, mechanical force, or electrical stimulation (Chen et al., 2020), rolling may also be achieved through the strategic design of the material properties of a substrate. For this transformation, a strain gradient is generated through the deposition of one or more materials, sometimes on a sacrificial layer, resulting in a multi-layer planar film. Once the sacrificial layer is degraded or mismatched swelling is induced, the film releases and rolls up like a jelly roll cake or a scroll. The resulting tubes are convenient structures for studying single to low numbers of cells and, based on the materials selected, can incorporate sensors.

**FIGURE 3:**
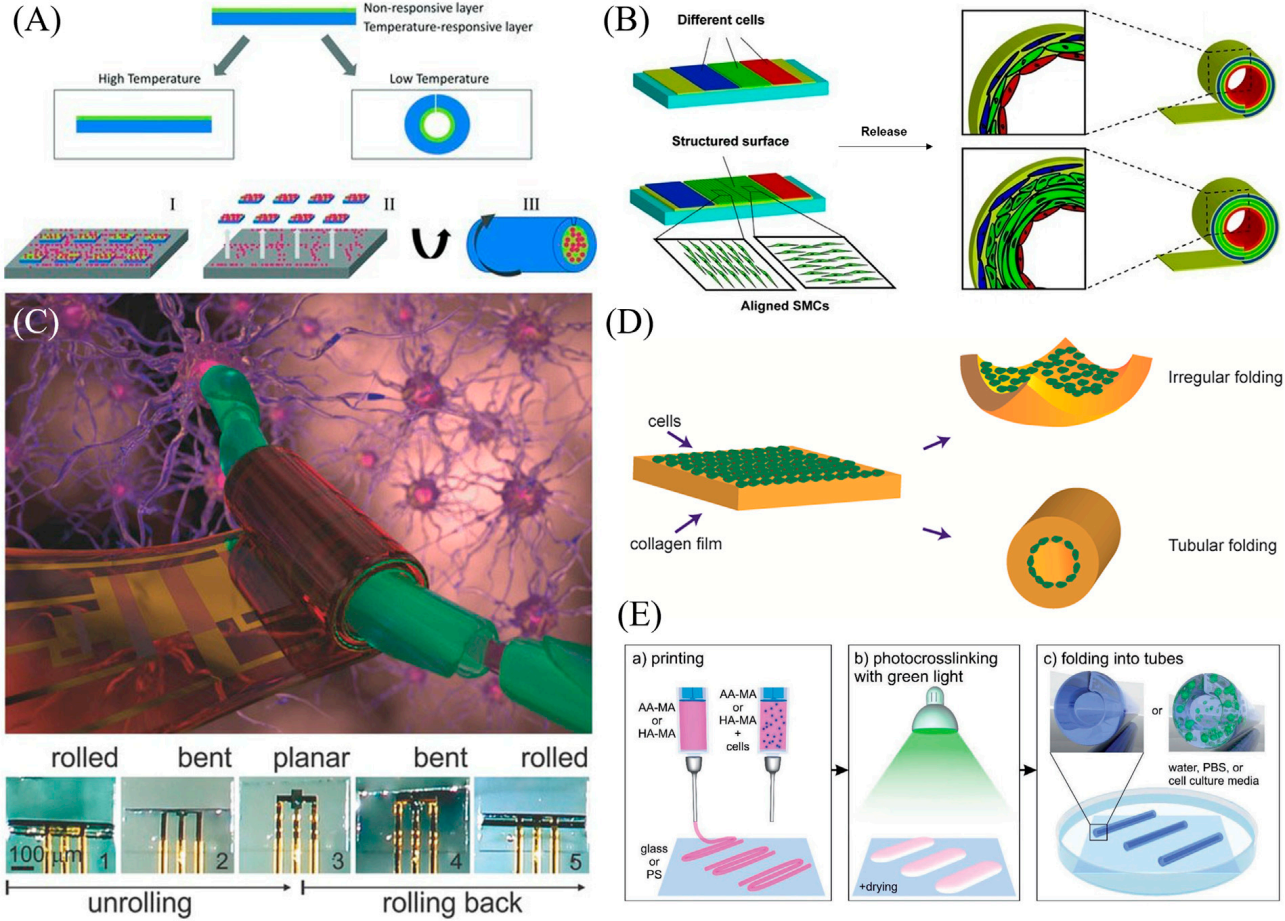
Examples of rolling shape transformation. **(A)** Cardiac progenitor cells are encapsulated and rolled based on a change in temperature leading to the swelling of a poly(N-isopropyl acrylamide) layer (Pedron et al., 2011). Reproduced with permission from (Pedron et al., 2011) © 2011 WILEY-VCH Verlag GmbH & Co. KGaA, Weinheim. **(B)** A stress-induced rolling membrane strategy allowed for the release of a polydimethylsiloxane film seeded with ECs, smooth muscle cells (SMCs), and fibroblasts to roll into a vessel-like structure (Yuan et al., 2012). Modified with permission from (Yuan et al., 2012) © 2012 WILEY-VCH Verlag GmbH & Co. KGaA, Weinheim. **(C)** Small circuits can be encapsulated within temperature-sensitive N-(2Hydroxyethyl)acrylamide and poly(ethylene-alt-maleic anhydride) bilayers that can roll up and enclose neural cells (Karnaushenko et al., 2015). Reproduced with permission from (Karnaushenko et al., 2015) © 2015 The Authors. Published by WILEY-VCH Verlag GmbH & Co. KGaA, Weinheim. **(D)** Cells within a collagen film actuate a shape change of their substrate. Directionality of the shape change was a function of collagen concentration, cell density, and cell contractility (Sang et al., 2017) Reproduced with permission from (Sang et al., 2017) © 2016 American Chemical Society. **(E)** A differential crosslinking gradient of a bioprinted cell-laden polymer monolayer allows for hollow tube formation (Kirillova et al., 2017) Modified with permission from (Kirillova et al., 2017) © 2017 WILEY-VCH Verlag GmbH & Co. KGaA, Weinheim.

Early rolled structures for biomedical applications featured each of the main actuation methods, stimuli-responsive polymers and release of sacrificial layers. A two-layer hydrogel was synthesized for the purpose of encapsulating cardiac progenitor cells, with the ultimate goal of delivering them to the myocardium by injection as a treatment for heart failure (Figure 3A) (Pedron et al., 2011). The film thickness varied from 20 to 40 μm prior to swelling and 300 μm–600 μm diameter upon swelling. The bilayer featured a temperature-responsive layer, poly(N-isopropyl acrylamide) (PIPAAm), and a nonresponsive layer, diacrylated triblock copolymer composed of poly(ethylene glycol) and poly(lactic acid) (PLA-b-PEG-b-PLA). The nonresponsive layer enabled cell adhesion, while the responsive layer allowed swelling-based actuation when the temperature was decreased below 37°C. The resulting structure was relatively transparent, and the shape change occurred on the order of a few hours. Encapsulated cells retained high viability, though some degradation of the polymer structure was observed over longer time frames (30–60 days).

Another early example using a polymeric bilayer has been used to encapsulate yeast (Zakharchenko et al., 2011). The resulting transparent film composed of polysuccinimide and PCL has the advantages of biocompatibility, biodegradability, and high porosity, allowing gas permeation. The diameter of the resulting tubes was controlled by the thickness of the films as well as by the degree of crosslinking, which was performed with photolithography. The shape transformation was induced by placing the films in room-temperature phosphate-buffered saline (PBS). This hydrolyzed the polysuccinimide to polyaspartic acid, which swelled and rolled the tube. Rolling of the films around the cells took 24 h, with swelling beginning at approximately 9 h. In contrast to many temperature-sensitive bilayers, this transformation is irreversible. There were also limitations in the control over the direction of rolling (i.e., rolling along the long side vs. rolling along the short side vs. rolling on the diagonal). Encapsulated yeast cells monitored up to 14 h after rolling showed promising viability based on proliferation behavior.

Tubular structures exist in the human body in different sizes and with different cell composition. Examples include blood vessels, the intestines, and the trachea. Thus, developing these structures is a crucial need in tissue engineering. To create tubular structures with multiple cell types, different cells were microfluidically patterned onto a thin PDMS membrane, which had been stretched and mounted onto a substrate using a layer of semi-cured PDMS (Yuan et al., 2012). The transformation to a rolled structure occurred using a stress-induced rolling membrane (SIRM) strategy wherein the film rolls up upon release from the substrate in approximately 1 minute’s time (Figure 3B). The thickness of the film could be tuned based on spin coating parameters, while the inner radius of the roll was a function of the tension on the membrane during the stretching process, ranging from 100 μm to 2 mm. Based on the design of the microfluidic cell patterning channels and the structure of the surface, different cell types can be patterned and aligned, and a vessel structure composed of ECs, smooth muscle cells (SMCs), and fibroblasts was constructed as proof of concept. While perforated PDMS allowed for oxygen and nutrient transport within the structure, imaging was limited due to the thickness of the PDMS, although the structures could be embedded and sectioned for image analysis.

Strain engineering was also used to enclose neuronal cells and mimic confinement by myelin sheaths (Froeter et al., 2014). Strategic photolithography and plasma-enhanced chemical vapor deposition were used to fabricate strained silicon nitride films on a sacrificial layer of germanium (Ge). Selective etching releases the film, which may be tethered in place through a photoresist “mesa.” The resulting rolls were 40 nm thick and ranged from 2.7 to 4.4 μm in diameter, designed to confine single neurons. Because the tubes were transparent, cells could be monitored by time-lapse confocal and phase contrast microscopy. The confined E15.5 cortical neurons showed high rates of growth cone outgrowth.

SiO/SiO_2_ transparent nanomembranes have similarly been used to confine cells for the study of cell division (Xi et al., 2014). The use of photolithography facilitated the fabrication of 500 “cavities” per 1 cm^2^ chip. The thin films were deposited on a sacrificial patterned photoresist layer. The films could be actuated through the degradation of the sacrificial layer using acetone, with rolling occurring within a few seconds of acetone exposure. The resulting tubes were then treated with Al_2_O_3_ to strengthen the structures, as well as fibronectin to increase the adhesion of the cells which migrated into the tubes. Based on the thickness of the films (range: 25–100 nm), tubes ranging from 4–20 μm in diameter could be fabricated. These structures were used to study mitosis and chromosome segregation errors in HeLa and RPE1 cells. This group has also used similar microtubes to mimic the behavior and proliferation of cancer cells within arterioles and microcapillaries (Xi et al., 2016) and fibronectin-functionalized glass microtubes to study different modes of cell migration using murine neural stem cells (Koch et al., 2015).

Work from this group has also incorporated microelectronics within a multilayer polymeric stack to guide and monitor neuronal cells (Figure 3C) (Karnaushenko et al., 2015). Structurally, a hydrogel swelling layer of N-(2Hydroxyethyl)acrylamide (HEAA) and poly(ethylene-alt-maleic anhydride) (PEMA) is layered on a polymeric sacrificial layer and reinforced with a polyimide film. Small circuits containing amplifiers and logic gates (Indium gallium zinc oxide (IGZO) transistors) were incorporated and protected with polychloroprene for a total thickness of under 1 μm, with the resulting rolled-up diameter being a function of the film thickness. Shape transformation occurred as isopropanol evaporated from the aqueous solution. The transparent structure enabled both imaging and preliminary steps toward electrical monitoring.

Cells themselves may also serve as the impetus for shape transformation, as shown in the formation of tubes from cells seeded in collagen films (Sang et al., 2017). Collagen containing cells was introduced to microfluidic PDMS channels and then gelled, during which time, the cells settled to the bottom of the collagen film. Upon release from the PDMS structures, the films rolled or puckered based on cell contractility (Figure 3D). Square films were 2 mm in side length and 200 μm in thickness, and rolling took approximately 3 days. The tendency of films to pucker vs. roll was a function of collagen gel concentration, cell density, and the contractility of the cells seeded. Additionally, the films could be structured with ridges for preferential rolling shape change. While this study focused on the mechanical and shape change properties of the cell-laden films, the materials and shapes achieved suggest future applications in tissue engineering.

Another transparent tubular structure allowed for the confinement of several cells to study neuronal behavior (Sakai et al., 2019). A bilayer nanofilm of graphene and poly(chloro-p-xylylene) (parylene-C) was deposited on a sacrificial layer of calcium alginate, which could be degraded with ethylenediaminetetraacetic acid (EDTA) following the addition of cells of interest. The rolling of the films into tubes with radii on the order of 10s of microns was induced and maintained by a combination of strain engineering and π-π adhesion between graphene and parylene-C. Rolling occurred over the course of 10 s to 2 min. The tubes could be rendered porous using reactive ion etching prior to cell introduction. The presence or absence of pores led to different behavior of the primary rat embryo hippocampal neurons used in this study. Cells in non-porous tubes aggregated, while porosity allowed axonal growth and distribution of neurons. Axons extending through the pores would potentially allow for connection with surrounding tissues while the cell body remains encapsulated, presenting potential tissue engineering applications.

Both gripping and rolling transformation may result based on the design of different swelling ratios within a transparent polymer substrate (Kwag et al., 2016). Photopatterned bilayers of poly(ethylene glycol) diacrylate (PEGDA) of differing molecular weights and PEGDA-methagel mixtures were fabricated within chambers on a substrate. Upon release from the glass, the films would curve based on differential swelling ratios, with the diameters ranging from 752.2–1,140 μm as a function of film thickness and ratio of components. The different resulting structures were intended to model ducts in the case of rolling and acini in the case of gripping. Breast cancer cells (SUM159 and MDA-MB-231) could be seeded or encapsulated and were viable for between 9 and 15 days, respectively.

While most of the previous studies used lithography to develop rolling structures, bioprinting has also been used to develop hollow tubular structures based on stimuli-responsive polymers. Kirillova et al. (2017) printed films of methacrylated hyaluronic acid and methacrylated alginate on glass and polystyrene as the substrate, and used visible green light for photocrosslinking (Figure 3E). In contrast to previous work in this field where two polymers with different swelling properties were required for bending deformation, the self-rolling property arose based on a crosslinking gradient through the depth of the film. Additionally, in the case of Ca^2+^-sensitive polymers such as alginate, the rolling/unrolling property could be reversed by increasing Ca^2+^ concentration using CaCl_2_ and decreasing its concentration using EDTA as a chelating agent. They showed that it was possible to tune the diameter of the resultant hollow tubes by changing the substrate, polymer concentration in the solution, printing speed, and crosslinking time. They were able to fabricate hollow fibers with an inner diameter of 20 μm. Compared to other extrusion-based bioprinting methods, this method was able to avoid printing smaller-diameter fiber and the resulting shear stress that often negatively affects cells within bioinks. They also printed cell-laden polymers and showed that it was possible to achieve self-rolling while incorporating cells within the material. Upon culturing cells for 7 days, cells were homogenously distributed through the tubes, and their viability did not significantly change from their initial printing. Accordingly, these hollow tubular structures could have applications in replicating neural networks, vasculature, and osteons, depending on the incorporated cells.

In summary, the rolled shape has been applied widely in different cell confinement and culture scenarios. The size and materials used may vary, with transparency enabling imaging. Cell incorporation may need to be performed strategically, depending on methods of actuation, such as how a sacrificial layer is degraded. Other considerations include how film thickness may negatively affect imaging quality, the effect of porosity on mass transfer (including gas exchange), and thus, the behavior and viability of encapsulated cells, and the ability to incorporate microelectronics for enhanced sensing capabilities.

### 3.2 Gripping

Strain engineering as well as differential material swelling have been used in the case of gripping structures, wherein planar shapes fold into a gripping shape through the closing of “flower petals” or “claws.” In addition to the examples described above, these studies yield structures that perform gripping motion upon various methods of actuation.

**FIGURE 4:**
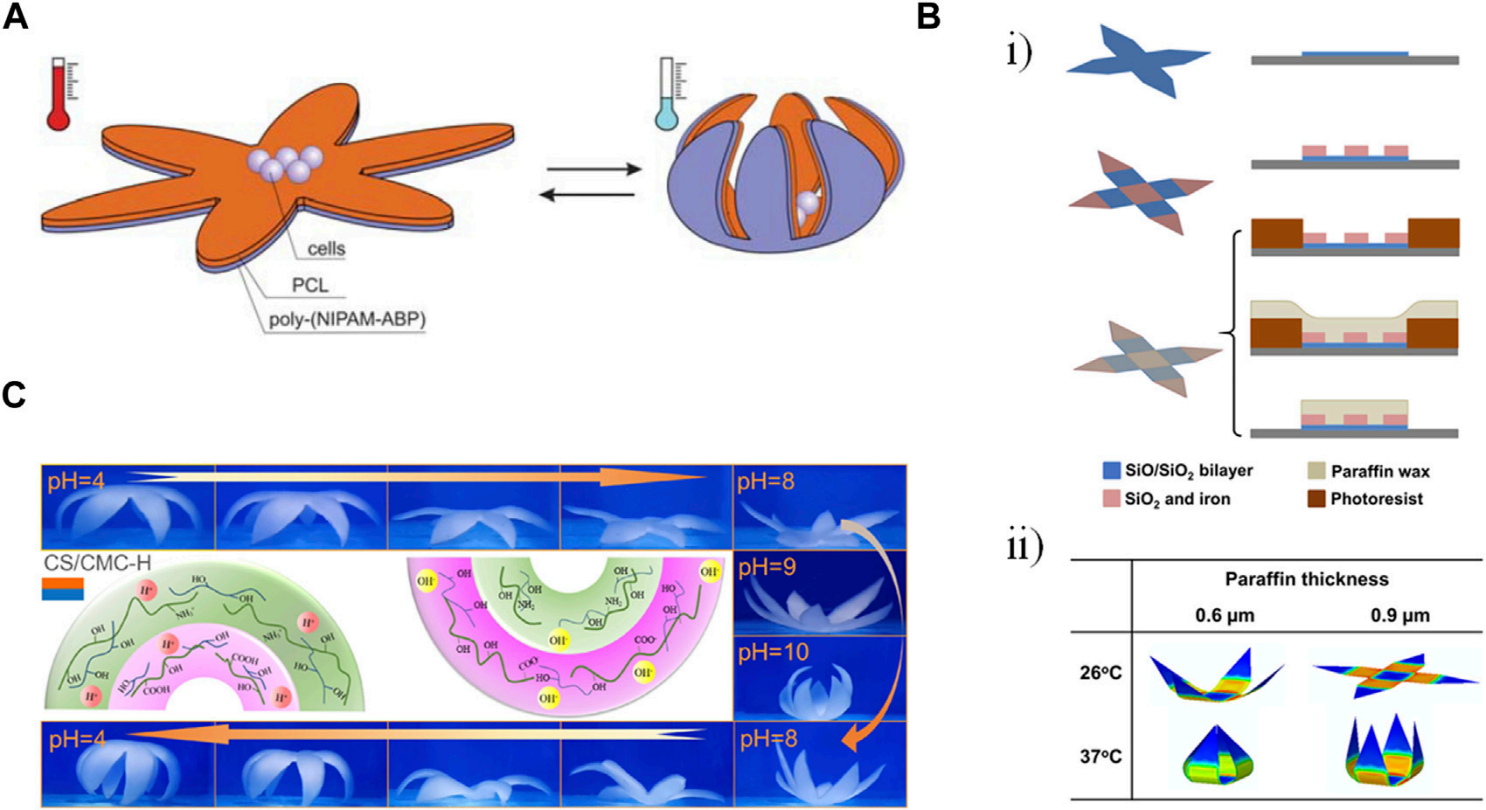
Examples of gripping shape transformation. **(A)** A star-shape bilayer of polycaprolactone (PCL) and poly-(N-isopropylacrylamide) copolymer containing 1 mol% of 4-acryloylbenzophenone comonomer (poly(NIPAM-ABP)). Decreasing the temperature below the lower critical solution temperature caused the swelling of the poly(NIPAM-ABP) layer, bending the arms and eventually closing the gripper (Stoychev, Puretskiy, and Ionov 2011). Reproduced with permission from (Stoychev, Puretskiy, and Ionov 2011) © 2011 The Royal Society of Chemistry. **(B) (i)** Fabrication of a thermally-responsive single-cell gripper in three steps: depositing a bilayer of silicon monoxide and silicon dioxide using lithography (top row), depositing a magnetically responsive rigid part containing silicon dioxide and iron (middle row), Patterning paraffin as the thermally responsive layer by molding (bottom row). **(ii)** Finite element simulation showing the effect of temperature and paraffine thickness on the folding angle of the gripper’s arms. Modified with permission from (Jin et al., 2020) © 2020 American Chemical Society **(C)** Pictures and mechanism of reversible deformation of a hexapetalous flower-like bilayer hydrogel composed of chitosan (CS) and carbomethyl cellulose (CMC) driven by swelling over a pH range (Zeng, Jiang, and Wu, 2023). Reproduced with permission from (Zeng, Jiang, and Wu, 2023) © 2023 American Chemical Society.

A controllable capture and release functionality was enabled through a gripping self-folding polymer microcapsules response to changes in temperature (Figure 4A) (Stoychev et al., 2011). Bilayers of PCL and PIPAAm were crosslinked together, leading to folding upon the swelling or shrinking of the PNIPAM layer due to changes in solubility at 33°C. Different star-shaped patterns folded up as the temperature decreased, with the degree of folding (i.e., incomplete folding vs. rolling from both directions) being a function of the relative thicknesses of the two films. While thicker PCL and thinner PNIPAM led to incomplete folding, the reverse led to multi-rolling. Films were on the order of 4 μm thick, allowing microscopy, and the structures had faces with side lengths of approximately 200 μm such that it was possible to encapsulate high numbers of yeast cells as proof of concept. With an actuation time of 5–10 s, this method of encapsulating cells has promise in the area of tissue scaffolds.

On a smaller scale, SiO/SiO_2_ bilayers have been patterned using photolithography to enable the high-throughput fabrication of single-cell grippers (Malachowski et al., 2014). Pre-stressed bilayers were assembled on a sacrificial copper layer, after which rigid regions of silicon monoxide were added to serve as hinges. The base of the gripper could be designed to either remain arrayed on the substrate or to be released. The bilayers ranged in thickness from 3–27 nm and were irreversibly actuated based on the degradation of a sacrificial layer in 37°C media over the course of 2–6 h. The bilayer grippers ultimately dissolved at 37°C over 20 days, releasing the captured cells. The use of photolithography allowed both high-volume fabrication (500,000–10 million grippers on a 3″ wafer) as well as the fabrication of different sizes of grippers with varying numbers of arms. The grippers were optically transparent, and the slits in the enclosed grippers allowed for the diffusion of nutrients, oxygen, and waste in and out of the structures. The grippers were able to capture mouse L-929 fibroblasts and beagle red blood cells; calcein-acetoxymethyl ester (calcein-AM) staining showed that the viability of the cells was not affected by the grippers.

Subsequent work by this group incorporated additional layers to increase control over the localization and folding of the grippers for potential applications in surgical biopsies (Jin et al., 2020). In addition to the stressed SiO/SiO_2_ bilayer, a magnetic layer comprising SiO_2_ and iron as well as a thermally responsive paraffin wax layer were added (Figure 4Bi). The SiO_2_/iron layer formed the hinges of the gripper while also allowing spatial control using magnets. The paraffin, which was spin coated over layers previously established using photolithography and then strategically lifted off, was rigid at low temperatures but melted with increasing temperatures. The folding angle of the gripper could be tuned based on the thickness of the paraffin layer (Figure 4Bii). While the bilayer film was transparent, the structures were largely opaque due to the iron in the hinges, making them more suited to cell manipulation rather than applications requiring imaging. Future work will also be needed to optimize timed actuation in the 37°C body as well as magnetic guidance in the presence of significant drag forces.

In addition to surgical applications, gripping structures can also be used for sensing functions (Zeng et al., 2023). Bilayer hydrogels consisting of different combinations of polysaccharides (anionic carboxymethyl cellulose (CMC), cationic chitosan (CS), and amphoteric carboxymethyl chitosan (CMCS)) were crosslinked with poly(vinyl alcohol) (PVA). The shape change could be actuated with changes in pH or humidity, with protonation/deprotonation of the various amine and carboxyl groups on the different polysaccharides, resulting in different swelling behaviors (Figure 4C). The SC/CMC-H bilayer combination had the most advantageous mechanical properties and was also mounted on a PDMS substrate containing copper wires such that gripping and releasing corresponded to changes in resistance. The sensors could be adhered to the cheek and throat of a human volunteer to sense blowing and swallowing motions.

The different gripping studies described above make use of different materials and actuation methods that ultimately affect their ultimate applications. Other gripping materials can be actuated with a change in pH (Yoon et al., 2014), which limits their actuation in the presence of cells. The use of sacrificial layers allows for thinner substrates but features longer actuation times. The scale of the resulting structures, as well as the conditions under which it is desirable that they actuate, must be considered in addition to other parameters, such as optical transparency, gas permeability, and biodegradability when selecting a strategy for a given application.

### 3.3 Folding

Although some of the above transformations enabled multiple 3D shapes encompassing folding as well as rolling and/or gripping, here we will provide some examples of folding that incorporate paper. Paper features numerous advantages, such as low cost, porosity (enabling gas exchange), flexibility, and fibrous structure (Lantigua et al., 2017). It can also be chemically modified and sterilized. However, paper can lose mechanical robustness when it becomes wet, though this can be mitigated with different coatings. The use of paper can yield relatively larger scale constructs, though they sometimes sacrifice the advantageous imaging properties of the hydrogels and thin films.

**FIGURE 5:**
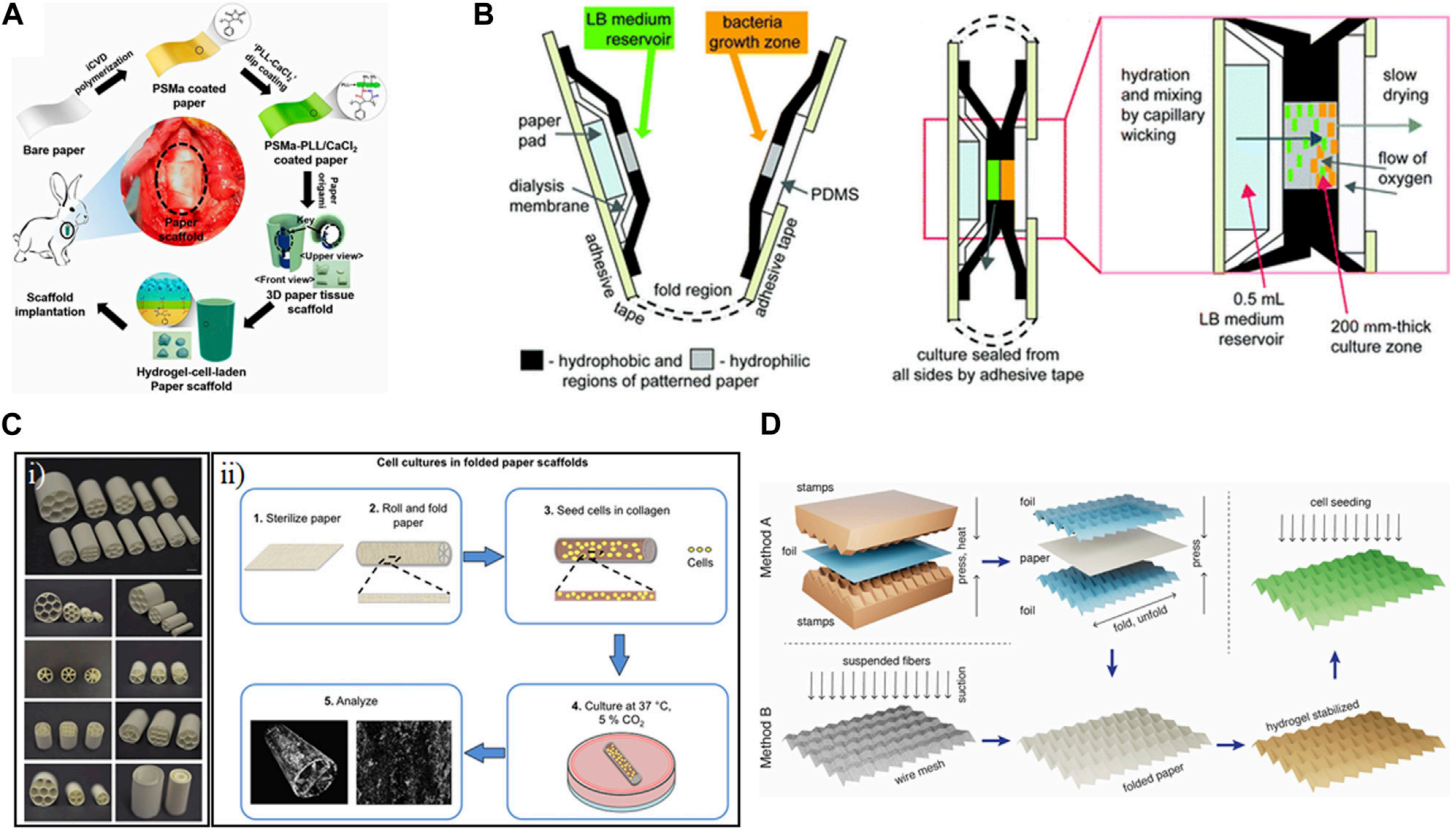
Examples of folding shape transformation using paper. **(A)** Paper coated with poly(styrene-co-maleic anhydride) (PSMa) using initiated chemical vapor deposition (iCVD) was dip coated in a solution of poly-L-lysine (PLL) and CaCl_2_. The resulting strengthened paper was then folded and immersed in a cell-gelatin solution. Hydrogel-cell-laden paper was transplanted into rabbits as a tracheal implant (Kim et al., 2015). Reproduced with permission from (Kim et al., 2015) **(B)** Macroscale folding resulted in a portable diagnostic device for bacterial culture and colorimetric readout (Funes-Huacca et al., 2012). Reproduced with permission from (Funes-Huacca et al., 2012) © 2012 The Royal Society of Chemistry. **(C) (i)** Rolled and folded cellulose paper was loaded with cell-laden collagen gel to be used as a tissue scaffold. **(ii)** Technique for shaping paper into a variety of structures and dimensions to fabricate individualized scaffolds (Camci-Unal et al., 2016). Reproduced with permission from (Camci-Unal et al., 2016) © 2016 The Authors. **(D)** Paper was molded into stretchable 3D Miura-ori patterns to serve as contractile scaffolds for cardiovascular tissue engineering (Rodriguez et al., 2023). Reproduced with permission from (Rodriguez et al., 2023) © 2023 The Authors.

Paper-based tracheal scaffolds on the order of several millimeters in diameter were constructed using coated paper, which could subsequently be folded into three-dimensional shapes (Figure 5A) (Kim et al., 2015). The initial paper layer was coated using initiated chemical vapor deposition of poly(styrene-co-maleic anhydride) (PSMa), which was then further immobilized with poly-L-lysine and calcium. The resulting strengthened paper could then be folded into different shapes, such as cylinders, and further coated with cell-laden alginate hydrogels. Upon hydration, the thickness of the walls of these structures ranged from 231 to 960 μm. The resulting structures, incorporating chondrocytes, were successfully implanted into rabbits.

Culture in the service of diagnostics was achieved using common-place materials in a colorimetric paper-based platform to monitor bacterial growth (Figure 5B) (Funes-Huacca et al., 2012). The devices were constructed in two dimensions, featuring paper patterned on either side to contain LB medium and a growth zone. The different papers were enclosed using packing tape and thin layers of PDMS. The device could be inoculated with a sample and then folded manually to bring the bacteria into contact with the media. By incorporating colorimetric components and calibration strips, bacterial quantification could be performed using a cell phone app. The device, which was on the order of several centimeters in size, was autoclavable. The result met the ASSURED criteria (Affordable, Sensitive, Specific, User-friendly, Rapid and robust, Equipment-free, and Delivered to those in need). Ultimately, this device had different goals than some of the other scaffolds, prioritizing functionality in low-resource settings over tunability and microscopy.

Commercially available paper has also been used to facilitate biomineralization on relatively large (centimeter-scale) structures (Camci-Unal et al., 2016). The results showed the trade-offs between 2D and 3D structures constructed using filter paper. The pure cellulose paper used was 190 μm thick with an average pore size of 25 μm. The paper could be folded and rolled into multi-compartment cylindrical scaffolds or could be left flat (Figure 5Ci). Murine osteoblasts were then seeded in a Collagen I gel (Figure 5Cii). The folded/rolled paper could not be imaged using microscopy, though there was some analysis with micro-computerized tomography. However, various staining analyses showed promising mineralization growing within the fibers of the flat paper.

Properties of paper, such as the alignment of its fibers and the tunability of its mechanical properties based on folding and wetting, can be exploited in the service of cardiovascular tissue engineering. Rodriguez et al. assessed different cellulosic papers, including cotton linters, eucalyptus fibers, pine, spruce, and lyocell, for their elastic moduli and tensile strengths, prioritizing those within the range found in cardiac tissue. Taking inspiration from origami, the 2D paper was folded into a 3D Miura-ori pattern with an assist from 3D printing (Rodriguez et al., 2023). Dry paper could be molded on foil that had been pre-configured using a 3D printed mold, while wet papers could be molded directed on 3D printed scaffolds (Figure 5D). The resulting structures, which were on the order of several centimeters in side-length, could be stabilized through encapsulation in a gelatin hydrogel. Initial concerns about potential biocompatibility issues due to the kraft bleaching process of the paper were allayed through viability staining of rat fibroblasts cultured on the structures, which, in the case of those cultured on cotton linters, showed higher viability than those cultured on tissue culture plastic. The long-term application of these paper structures with cardiomyocytes could lead to additional cell-actuated shape change due to contraction, making it a promising structure for applications in *in vitro* models and regenerative medicine.

Ultimately, the larger-scale structures resulting from origami-inspired folding show promise due to their easy-to-acquire components and may be beneficial in tissue engineering applications if limited in the context of performing biological studies.

## 4 Biological applications of shape-morphing materials

An inspired use of shape-morphing materials is their application in developing biological systems (Table 3) as they can mimic the dynamic nature of the response of such systems to environmental stimuli. Additionally, they can simplify the fabrication of complex devices, and their shape-changing property can overcome challenges resulting from the complexity of biological structures.

**TABLE 3:** Summary of biomedical applications of shape-morphing structures.

Biomedical area	Application	Requirements	Transformation	Material	References
Minimally invasive devices	Intestinal anastomosis	• Precise joining of two bowel ends without tension	2D to 3D	PVA/gelatin	Paonessa et al. (2017)
		• Reducing post-operation inflammatory reactions	3D to 4D	PLA/PLGA	Peng et al. (2023)
		• Avoiding anastomotic leakage			
		• Mitigating inflammation and tissue damage caused by foreign body objects			
	Biopsy	• Miniaturized and untethered device for retrieval of tissue from limited-access sites	2D to 3D	Cr/Cu/Ni/Au/Microposit SC 1827	Leong et al. (2009)
		• Remote actuation and directing of grippers	2D to 3D	PCL/p(NIPAM-ABP)	Stoychev, Puretskiy, and Ionov (2011)
		• Selective closure triggered by specific cues	2D to 3D	Cr/Cu/Ni/Au SC1805/SC1813	Gultepe et al. (2013)
		• Appropriate gripping capability	2D to 3D	pNIPAM-AAc/PPF/Fe_2_O_3_	Breger et al. (2015)
		• Feasible and high-rate removal strategy of the grippers	2D to 3D	P(OEGMA-DSDMA)/P(AAm-BAC)/Fe_2_O_3_	Kobayashi et al. (2019)
		• High cell viability percentage after being captured in the gripper	2D to 3D	SiO/SiO_2_/iron/paraffin wax	Jin et al. (2020)
		• Optical transparency			
		• Biocompatible and biodegradable materials			
		• Biocompatible actuation method			
		• Reversible actuation method			
Tissue Scaffolds	Single cell study in spatially confined tubular structure	• Tubular confinement of a single cell	2D to 3D	SiO/SiO_2_/fibronectin	Xi et al. (2014, 2016), Koch et al. (2015)
		• Transparency			
		• Tunability of the diameter of the structure			
		• Adhesiveness of the substrate			
		• Biocompatible rolling mechanism			
		• Biocompatible materials			
	Tubular scaffolds	• Biocompatible rolling mechanism	2D to 3D	silk fibroin hydrogel/parylene-C/Ca-alginate	Teshima et al. (2017)
		• Biocompatible materials	2D to 3D	Hyaluronic acid/Alginate	Kirillova et al. (2017)
		• Proper mechanical properties			
		• Tunability of the diameter of the structure			
		• Internal diameter as low as 8–20 μm			
	Hydrogel for enteroatmospheric fistula closure	• Avoiding leakage of intestinal fluid	3D to 4D	AAm-AAc-Fe^3+^/CNC	Qu et al. (2022)
		• Fitting in the bending structure of bowel			
	Cardiac construct for myocardial regeneration	• Myoblast alignment	3D to 4D	bisphenol A diglycidyl ether/poly(propylene glycol) bis(2-aminopropyl) ether/decylamine/graphene	Wang et al., 2021
		• Synchronized beating of the cardiac tissue			
	Implant for improving bone healing by regulating stem cells	• Wrapping the complex geometry of the defected area	3D to 4D	PCLDA1000/PCLDA2000/BPO/PSPMA/Irgacure 819	You et al. (2021)
		• Modulating the micro-structure of the scaffold to promote either proliferation or differentiation of stem cells			
Sensor	Stretchable sensors	• Integration of analytical, and sensitive electronic devices	Stretch in 2D	Parylene-C/Titanium/Platinum	Morikawa et al. (2018)
		• High stretchability	2D to 3D	Parylene-C/Gold/	Morikawa et al. (2019)
		• High deformability			
	Wearable sensors	• Integration of deposable analytical, and sensitive electronic devices	Stretch in 2D	Paper/graphene/polyurethane	Gao et al. (2018)
		• Low cost	Stretch in 2D	Polyimide/Cupper/silicone rubber	Yamagishi et al. (2019)
		• High flexibility to avoid restricting natural body movement	2D to 3D	DNA strands/Cy5/BHQ3/glucose oxidase	(Li et al., 2023)
		• Ability to detect physiological indicators			
		• Minimizing invasiveness			

### 4.1 Minimally invasive devices

Medical devices are defined as tools, equipment, implants, or *in vitro* substances with the purpose of diagnosing or treating diseases within the human body (Gómez-Mascaraque, 2014). The invasive medical procedures required for implantation can have repercussions such as hospitalization, the need for anesthesia and blood transfusion, and prolonged postoperative recovery (Raucci et al., 2020). Minimally invasive surgery can mitigate these negative effects by inserting devices through smaller access openings, resulting in reduced surgery time, minimized tissue trauma, better cosmetic outcome, and reduced hospitalization (Zadeh and Daftary, 2004; Shah and Shah, 2008).

Shape-morphing materials are strong candidates to create efficient implants and devices for minimally invasive surgery because such structures can be introduced to the human body through a small incision and, upon applying a stimulus, result in a structural change to facilitate function at the required site. For shape-changing polymers, the wide range of glass transition temperatures and elastic moduli can be modulated to suit the desired application. Light weight and low cost are considered other advantages of these materials over traditional materials for biomedical purposes (Sokolowski et al., 2007; Zhao et al., 2019; Xiao and Huang, 2020). Criteria and required properties for specific applications are discussed elsewhere (Chan et al., 2016; Xiao and Huang, 2020).

#### 4.1.1 Anastomosis

Anastomosis is the procedure of connecting two vessels in the human body. Traditionally, a suture-based method has been used, which entails hand sewing both ends of two vessels or a vessel to a synthetic graft. It could potentially lead to scarring, tissue necrosis, and wound infection. Other disadvantages include time-consuming operation and the dependency on the dexterity of the surgeon for a successful surgery, specifically in the case of small vasculature, (Mallela et al., 2021). Sutureless anastomosis techniques have been developed to overcome the challenges and limitations of manual suturing, which could reduce the time and invasiveness of surgeries. Methods such as the Murphy button and magnetic compression have been developed but have drawbacks such as post-operation inflammation or the challenging process of inserting a ring into the human body (Jose et al., 2015; Zhao et al., 2019). To avoid these side effects, a biofragmentable anastomosis ring method could be implemented, which uses polyglycolic acid that will be resorbed once the healing process is completed. However, the insertion of the ring in the human body is invasive (Bobkiewicz et al., 2017; Peng et al., 2023).

**FIGURE 6:**
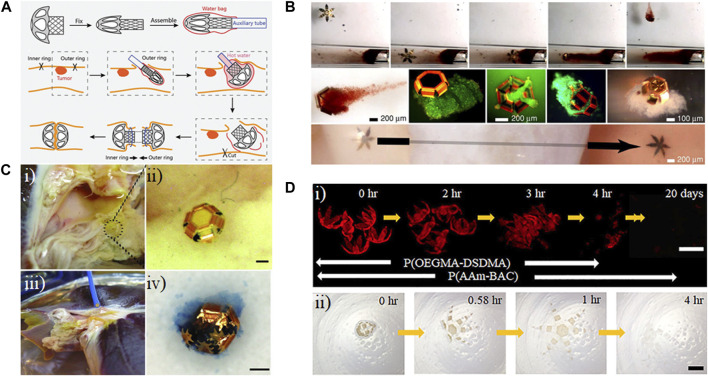
Applications of shape-morphing materials in minimally invasive devices. **(A)** Assembly and intestinal anastomosis process for a temperature-sensitive, 3D-printed polylactic acid ring (Peng et al., 2023). Reprinted with permission from (Peng et al., 2023) © 2023 Wiley-VCH GmbH. **(B)** Remote guidance of a tetherless, temperature-sensitive microgrippers within a tube using a magnet (top row). Live/dead staining of captured over the course of 72 h (middle row, green indicated viable). A microgripper traverses a bovine bladder sample (bottom row). (Leong et al., 2009). Modified with permission from (Leong et al., 2009) © 2009 The National Academy of Sciences of the USA. **(C) (i)** and **(ii)** Microgrippers were applied to retrieve cells from a porcine liver. **(iii)** A magnetic catheter is used to retrieve the microgrippers. **(iv)** Image of a retrieved microgripper with the excised tissue inside, stained with trypan blue. Scale bar represents 100 μm. (Gultepe et al., 2013). Modified with permission from (Gultepe et al., 2013) © 2013 WILEY-VCH Verlag GmbH & Co. KGaA, Weinheim. **(D)** Degradation of polymeric bilayer grippers in 50 mM glutathione solution (GSH) **(i)**, and 500 mM ascorbic acid (vitamin C) and 500 mM hydrogen peroxide at pH = 3 **(ii)**. Poly(oligoethylene glycol methyl ether methacrylate-bis(2-methacryloyl)oxyethyl disulfide) (P(OEGMA-DSDMA); poly(acrylamide-N,N′-bis(acyloyl)cystamine) (P(AAm-BAC)) (Kobayashi et al., 2019). Modified with permission from (Kobayashi et al., 2019) © 2018 American Chemical Society.

One approach to reducing the invasiveness of anastomosis devices is the use of shape-changing materials. Peng et al. (2023) used 3D printing to develop a 4D printed structure for sutureless anastomosis. To develop this structure, they tested different ratios of PLGA and PLA. Adding PLGA to PLA led to increased shape recovery compared to PLA due to reduced cold crystallization effect, while after a threshold, it had the opposite effect due to low crystallinity of the PLGA. The 1:1 ratio of the polymer had proper shape recovery, but the glass transition temperature was around 60°C, which was incompatible with biological application. To overcome this problem, acetyl tributyl citrate was added to the blend to reduce the glass transition temperature, making the blend biocompatible. This blend was 3D printed to create inner and outer rings. To provide heat for the change in shape, the rings were placed in acid-soluble chitosan bags injected with hot water. Inside the intestine, the shape recovery of the rings will occur, the bag will be degraded, and anastomosis will take place through snap-locking of the expanded rings (Figure 6A). Another important feature of this device is degradation. Based on degradation in PBS, there were holes in the structure after 5 weeks, and it eventually broke into fragments. However, it is likely that this structure would degrade faster in the human body compared to *in vitro* since lipid and biological components *in vivo* increase the polymer chain mobility (in which segments of the polymer rotate in different directions) and act as plasticizers (which increase the water uptake of the scaffold). Additionally, the autocatalytic effect of acidic byproducts is minimized *in vitro* due to media changes, which would not occur *in vivo* (Tracy, 1999; Lu et al., 2000). As a result, this degradation rate is suitable for its application; while the structure supports tissue reconstruction, it will not stay in place for longer than necessary, which could lead to unwanted inflammation. Another advantage of this structure was that the 3D printing used for its fabrication allows for customization based on patient size.

#### 4.1.2 Biopsy

Another area of research in minimally invasive surgery is the development of grippers to perform biopsies. A shortcoming of current minimally invasive grippers is that low pinch force could lead to loose gripping and the tissue potentially slipping out of the gripper. On the other hand, high pinch forces could cause unwanted tissue damage (Kortman et al., 2023). Most currently used grippers are tethered and lack the direct and fine-tuned control necessary to grip cells or other objects in coiled environments and small vasculatures (Dunn et al., 2022).

To address such issues, Leong et al. (2009) developed a system for multilayer untethered thermobiochemically actuated microgrippers. They used PVA as the sacrificial layer to allow the release of microgrippers from the substrate. First, thermal evaporation was used to pattern thin layers of chromium and copper on top of the PVA layer. Next, photolithography was used to pattern first nickel and gold for phalanges, then the polymer that triggers the shape change. Their preliminary studies showed that a gripper with six digits and three joints could have a proper gripping and shape-changing properties, i.e., the ability to completely close the structure and grip an object. The tip-to-tip size of the grippers in the open and closed states were 700 μm and 190 μm, respectively. The use of nickel in the structure enabled the remote control of the untethered microgrippers (Figure 6B). It was possible to trigger the closure of the structure using temperature and biochemical components such as L929 cell media. The shape-changing property of the grippers was not reversible; therefore, Pasteur pipettes, syringe tips, or vortexing were used to open the gripper and retrieve the cells. To perform an *in vitro* biopsy, they placed a bovine bladder in a capillary glass and guided a microgripper through the bladder. Then, the sample was heated to close the gripper, and a magnet was used for the rotation of the gripper, enabling it to isolate a cell mass from the tissue connected through extracellular matrix (ECM).

While the *in vitro* study showed promising results, it cannot ensure successful results for *in vivo* biopsy due to the presence of muscular motion, blood and body fluid flow, more difficult visualization, and constraints in inserting and retrieving the grippers. Accordingly, Gultepe et al. (2013) also developed microgrippers in a similar manner that were 350 μm to 1.5 mm to perform *in vivo* biopsy. They used copper as the sacrificial layer and chromium as the stressed thin film. The rigid parts of the structure were composed of nickel and gold. For the trigger polymer, a 5:1 mixture of Microposit SC1805 and SC1813 was used to control the transition temperature and the time for the actuation based on the intrinsic properties of the chosen polymers. At 4°C, these structures were open, while upon being heated to 37°C, they reached complete closure in 10 min. They applied these microgrippers for *in vivo* to showcase their ability to biopsy in areas that are not easily accessible (Figure 6C). Using a magnetic catheter, they were able to retrieve 95% of the microgrippers from the bile duct from the porcine liver. To demonstrate the quality and quantity of the excised tissue, they showed that they were able to extract sufficient DNA to perform PCR.

One problem with the above students was that the actuation was irreversible, increasing the difficulty of retrieval of cells or tissue. In contrast, Breger et al. (2015) developed microgrippers that could undergo reversible actuation. For this purpose, they developed a bilayer hydrogel from poly(N-isopropylacrylamide-co-acrylic acid) (pNI-PAM-AAc) and polypropylene fumarate (PPF). It is possible to tune the biodegradation rate of (pNI-PAM-AAc) based on the type and the density of crosslinking. The main products of PPF’s degradation are products of Krebs’s cycle; therefore, a structure based on these two polymers is biodegradable. Since both polymers were photopatternable, sequential lithography was used, enabling high-throughput production of these hydrogel-based microgrippers. For remote guidance of the microgrippers, they also incorporated iron oxide nanoparticles. The first step to grasp cells began at low temperatures, with the grippers closed with the PPF on the inside. Once they were transferred to a culture of fibroblasts at an increased temperature, the grippers opened. After gripping the cells, they closed with the PPF layer facing outside. The transparency of the arms allowed fluorescent visualization of cells, and staining with calcein-AM showed that cells live cells inside the grippers.

Microstructure biocompatibility and biodegradable is another important issue for their *in vivo* applications since 100% retrieval of microgrippers may not be achieved. Kobayashi et al. (2019) developed biodegradable microgrippers consisting of poly(oligoethylene glycol methyl ether methacrylate-bis(2-methacryloyl)oxyethyl disulfide) (P(OEGMADSDMA)), as the swellable part for triggering shape-change, and poly(acrylamide-N,N′-bis(acyloyl)cystamine) (P(AAm-BAC)) as the stiff part to improve gripping. Since both polymers were photopatternable, two-step photopatterning was used to create the grippers, and they could be released from the alginate sacrificial layer using deionized water or 1x PBS. Similar to the previous work from this group, they used iron oxide for navigating the grippers remotely. The presence of disulfide bonds in both of these polymers ensured biodegradation in the presence of disulfide-reducing agents (Figure 6D), and since the byproducts were mostly uncrosslinked polymers rather than monomers, the degradation was considered safe compared to monomer-producing degradation. To prove its biodegradability, they showed that the grippers were degraded in the presence of glutathione at its extracellular concentration range. Biocompatibility was displayed by culturing hTERT HAEC cells in the presence of the grippers for 3 days, after which 70% of cells in contact with the substrate remained viable. To assess the gripping property, they heated the structure to be opened then guided it toward a target object by remote controlling using a magnet. Once the grippers reached their destination, they decreased the temperature below the transition temperature to close the structure such that it gripped the target. After that, the gripper was transferred to another site, and heating was used to open the structure and release the target object.

### 4.2 Tissue scaffolds

In the human body, the ECM surrounds cells and provides them with a dynamic, interactive 3D environment with biological, mechanical, and chemical cues. Additionally, cells can also affect the properties of the surrounding environment (Brooks et al., 2022; Mirzababaei et al., 2023). For instance, a study in liver cancer showed that cancer cells can increase the porosity of the scaffold by up to 14% compared to healthy liver cells (Ozkan et al., 2019). Accordingly, it is imperative to develop scaffolds for a specific tissue/organ to study this reciprocal interaction.

While the dynamic response of biological tissues to environmental stimuli is an important area of investigation, most earlier studies are based on rigid biomaterials. This makes them unable to mimic the conditions cells experience in the human body, which is a challenge in the field of tissue engineering (Qu et al., 2023). This is significant for tissues such as the lung, heart, and muscle, where functionality depends on the elasticity and flexibility of the tissue. With advances in the field of material science, developing scaffolds and implants based on shape-morphing materials has been proposed as a method to avoid the complexity of previous fabrication methods and to make scaffolds and implants with dynamic properties (Holman et al., 2021). These scaffolds could be used for tissue regeneration or repair, implants, or biomaterials for culturing cells *in vitro*. The common theme in all of these applications involves the use of time as the fourth dimension, which allows the scaffolds to change their appearance or function in response to environmental stimuli, resulting in dynamic behavior (Zhang et al., 2023).

#### 4.2.1 *In vitro* models

*In vitro* models, a tool to study cells outside the human body, have been developed for purposes such as studying cell and tissue function and drug screening. In traditional cell culture, cells experience a 2D rigid environment in the form of tissue culture plastic, which is different from their 3D environment in the human body (Mirzababaei et al., 2023). Accordingly, biomaterial-based *in vitro* models such as cell-loaded electrospun micro/nanofibers, bioprinted scaffolds, organ-on-chip models, etc. have been developed to mimic the microenvironment of specific tissues and evaluate their interactions with biomaterials (Szabó et al., 2023).

**FIGURE 7:**
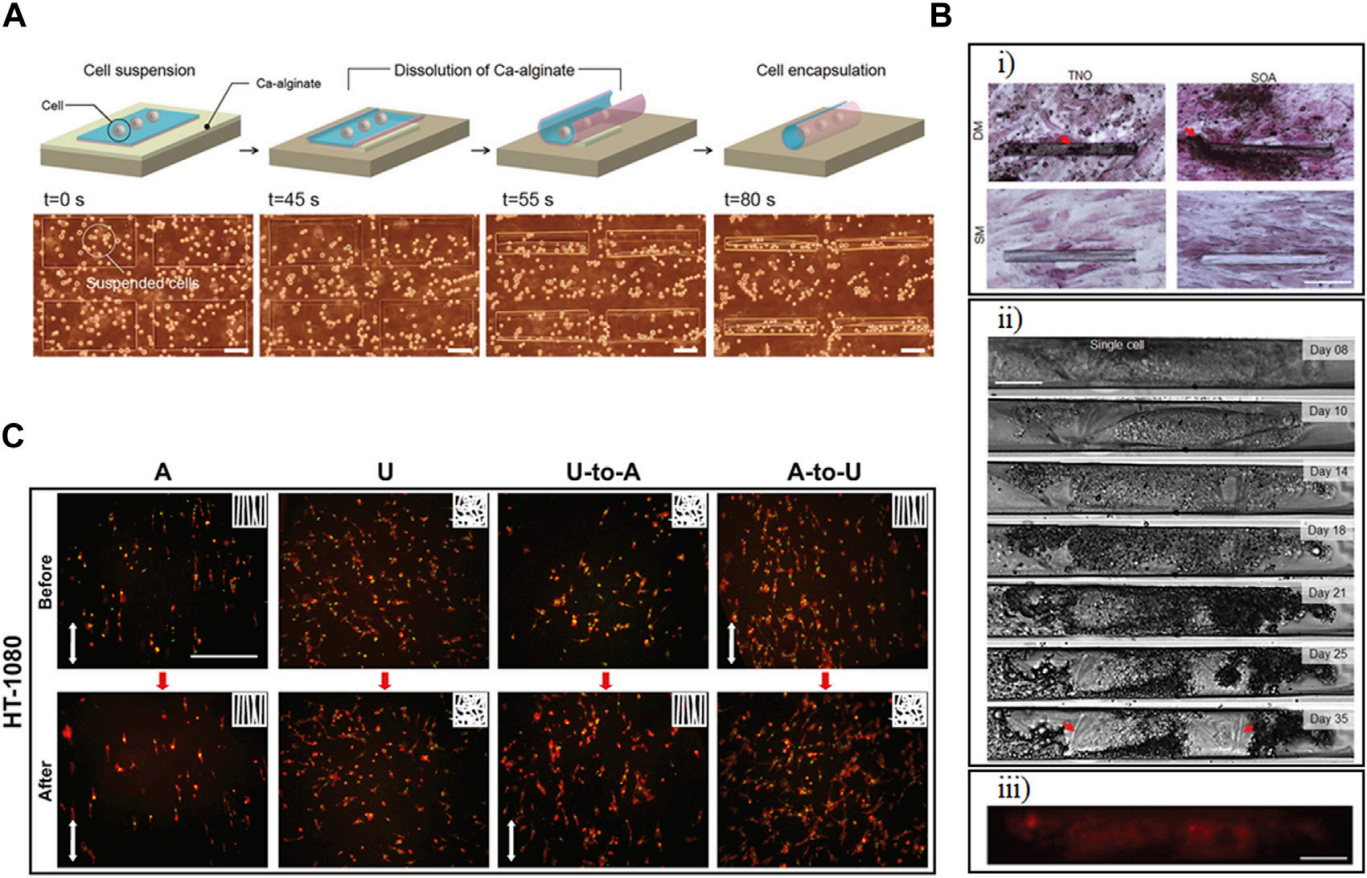
Developing *in vitro* models using shape-morphing materials. **(A)** Rolled films of polymer layers, silk fibroin, and parylene-C formed scaffolds to model characteristic cell behavior exhibited by endothelial cells (ECs) and hippocampal cells. Scale bar represents 100 μm. (Teshima et al., 2017). Reprinted with permission from (Teshima et al., 2017) © 2017 The Authors **(B)** Mesenchymal stem cells (MSCs) modeled varying degrees of differentiation behavior when incorporated in (Ti_x_Nb_1−x_)O_2_ (TNO) or SiO/SiO_2_/Al_2_O_3_ (SOA) tubular scaffolds and cultured with either standard medium (SM) or differentiation medium (DM). **(i)** von Kossa staining for calcium deposition, an indicator of biomineralization, after 3 weeks of culturing cells inside tubular structures in DM. Scale bar represents 100 μm. **(ii)** Single cell observation and **(iii)** live/dead staining of the cells within the scaffold. The scale bars for **(ii)** and **(iii)** represents 25 μm. (Herzer et al., 2021). Modified with permission from (Herzer et al., 2021) © 2021 The Authors. Small published by Wiley-VCH GmbH. **(C)** Studying the effect of the microstructure of tissue scaffolds on cells using shape-morphing materials. Cell Body (red) and nuclear (green) staining of HT-1080 cells cultured on aligned **(A)**, unaligned (U), switching from U to A and A to U scaffolds, showing increased preferential alignment of cells on A or dynamic scaffolds with thermally triggered from U to A. Scale bar represents 200 μm (Wang et al., 2017). Reprinted with permission from (J. Wang et al., 2017) © 2017 Elsevier Ltd.

Developing 3D tubular structures with tunable diameter is of interest in tissue engineering due to the diverse tubular structures found in the human body. Teshima et al. developed multilayer, self-rolled tubular structures based on strain engineering as a high-throughput method for manufacturing such structures. In this method, the polymer layers, silk fibroin, and parylene-C were coated onto a sacrificial layer, calcium alginate, selected such that cells could be seeded and the actuation would be biocompatible. The calcium alginate could be degraded based on using EDTA, rolling up and releasing the films on the order of tens of seconds (Figure 7A). Different dimensions and designs allowed for various rolling and gripping transformations, with the radii of the tubes related to film thickness and length and width parameters. The transparent nature of the film enabled visualization of ECs, hippocampal cells, and cardiomyocytes used in proofs-of-concept. Cells showed characteristic behavior, such as the formation of microvasculature by the ECs and dendrite extension by the hippocampal cells.

The fate of stem cells is important in regenerative medicine and cell therapy. Stem cell interaction with their environment can affect their proliferation, migration, and differentiation (Khan et al., 2024). Smart materials have been developed to investigate how their shape-changing properties could affect stem cell behavior. Herzer et al. (2021) developed a tubular structure to mimic specific biological materials as in a tubular scaffold of β-stabilized alloy Ti-45Nb, which is close to the elastic modulus of bone. These transparent scaffolds, while rolled up based on the degradation of a Ge sacrificial layer, were then used to study osteogenic differentiation, migration, and adhesion of MSCs at single-cell resolution in curved structures (Figure 7B). The tubes were porous, allowing gas transfer, and ranged from 12–30 μm in diameter and cells were maintained for up to 5 weeks, though by that point the cells had outgrown their tubular scaffolds. Their results showed that the tubular structures that mimics the diameter of the MSC’s microenvironment was able to improve proliferation and cell adhesion. The tubular structure, also, resulted in a change in the spindle-like morphology of MSCs on planar surface to a more elongated shape. By live study of the cells, they showed that cells initially identify the entrance and then migrate within the tubular structures. Their study also showed that migration mode changed from mesenchymal (elongated morphology) to amoeboid mode (rounded morphology) in the case of 3D tubular structures.

In a bone-related application, a graphene-cellulose paper has been used to support and differentiate adipose-derived stem cells (ADSCs) (Li et al., 2019). Many widely available papers were used, including Kim wipes and Scott tissues. The paper was coated in a graphene oxide solution that was subsequently converted to reduced graphene oxide (RGO) using ascorbic acid. Depending on this initial paper and the amount of graphene oxide per round of coating, thicknesses ranged from 51.6 to 372.0 μm, and conductivity increased with rounds of coating, ultimately leveling off once the paper became saturated. Three-dimensional structures on the order of hundreds to thousands of microns could be created by rolling or folding the paper laminated with cells encapsulated in an alginate hydrogel. The constructs were monitored over the course of 7 days for proliferation, Alkaline phosphatase activity, and mineralization, though imaging was conducted on cross-sections rather than the intact structure. These essays showed the improved ability of these 3D RGO-coated cellulose scaffolds for osteogenic differentiation of ADSCs compared to 2D structures.

Aside from a change in the macrostructure of a scaffold, shape-changing materials could be used to study how microstructure affects cells. Wang et al. (2017) developed electrospun fibers from thermoplastic polyurethane with thermal control over the alignment of the fibers. Both the fixing ratio and recovery ratio were 99%, and the scaffold showed 99% recovery at 37°C in 5 h. They observed that an increase in temperature led to an increase in the alignment of the fibers and that the polarized motility of the human fibrosarcoma cell line HT-1080 increased as well. The change in the alignment also affected the morphology of the cells. Upon thermal triggering and increased alignment of the fibers, cells, and nuclei elongated in the direction of the fibers, showing that the electrospun fiber has the capability to control the alignment of the cells (Figure 7C). This aligned arrangement of the cells could be useful in developing cardiac tissue with synchronized beating and neuron-like tissues.

#### 4.2.2 Implants

Transplanting a tissue or an organ from a donor to patient is a strategy to manage tissue loss or organ failure. However, donor shortage continues to challenge the healthcare system, motivating the development of artificial tissues and organs (Platt and Cascalho, 2022). Most of the previous biomaterial-based artificial organs fail to mimic the dynamic features of biological tissues. Shape-morphing materials could enable the complex structures required to recapitulate such features.

**FIGURE 8:**
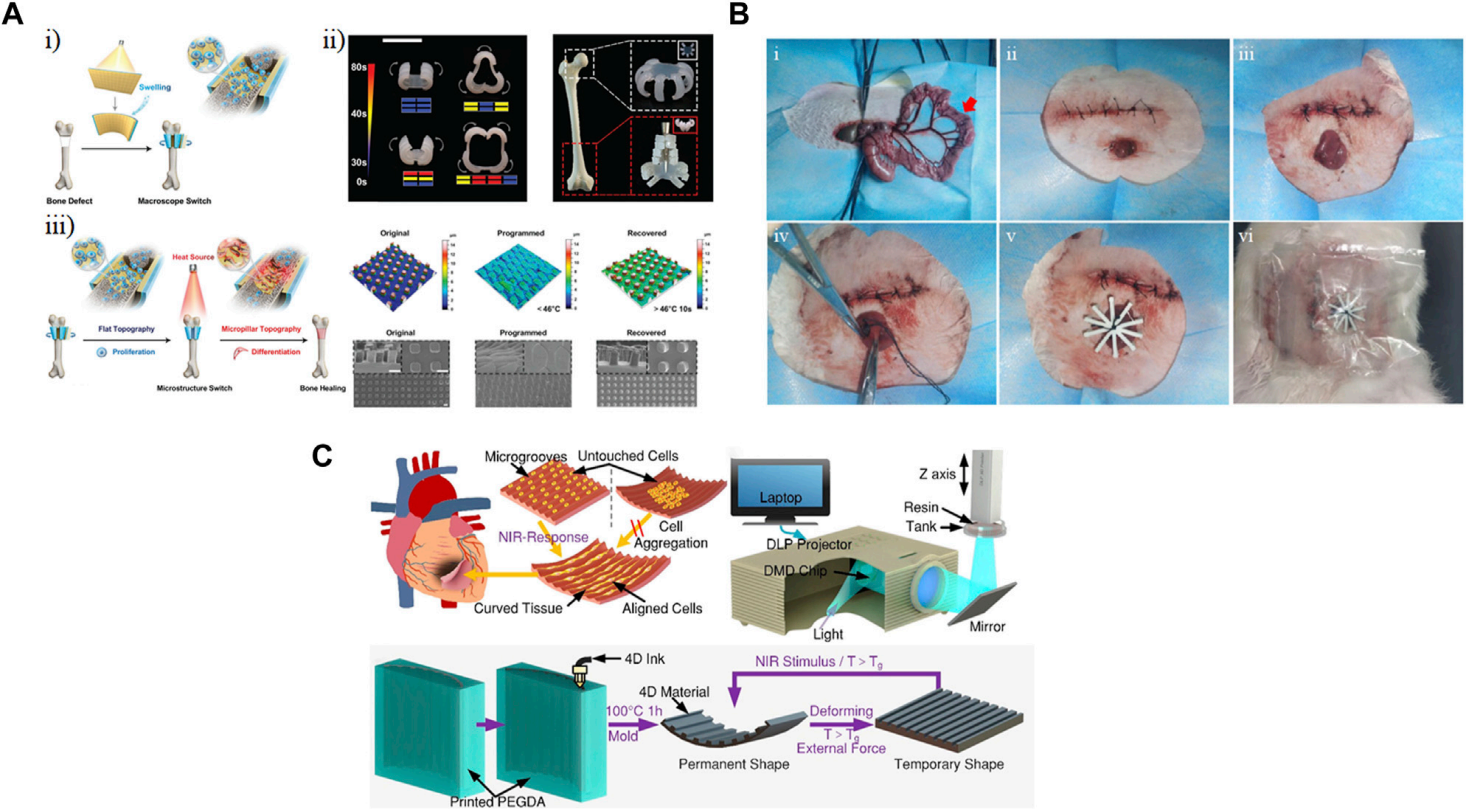
Implanted shape-changing materials. **(A)** Hydrogels comprising poly(ε-caprolactone)-diacrylates (PCLDA), 2-hydroxy ethyl acrylate (HEA), and 3-sulfopropyl methacrylate potassium salt (PSPMA) were printed using digital light processing (DLP). **(i)** Shape change occurred based on polymer swelling, and **(ii)** different shapes and patterns were designed to fit different bone regions. **(iii)** Modulating cell fate of mesenchymal stem cells (MSCs) by reversible change in the topography of the scaffold. Scale bar represents 5 mm (You et al., 2021). Modified with permission from (You et al., 2021) © 2021 Wiley-VCH GmbH. **(B)** 4D-printed bilayer hydrogel was implanted to seal an enteroatmospheric fistula in a New Zealand white rabbit model (Qu et al., 2022). Reprinted with permission from (Qu et al., 2022) © 2022 The Authors. Published by Elsevier Ltd. **(C)** A 4D printed scaffold could be used to both align relevant cell types and conform to the curvature of the heart as actuated through near-infrared (NIR) light (Wang et al., 2022). Modified with permission from (Wang et al., 2021) © 2021 American Chemical Society.

Bone defects have life-long effect on patients’ quality of life and how they function. Proliferation of MSCs followed by their differentiation towards osteoblasts is required for bone repair. The topography of a scaffold significantly impacts the cell fate of an MSC. While a smooth surface supports cell adhesion and proliferation, micro-structured patterns on the surface favor differentiation. You et al. (2021) used a digital light processing printer to fabricate a bilayer membrane, comprising a one hydrogel layer and a shape-memory polymer, the microstructure of which could be tuned by temperature. Although the structure was printed in 2D, swelling of the hydrogel resulted in a change in the macrostructure of the bilayer membrane (macroscopic switch), enabling it to wrap the bone defect (Figure 8Ai). They showed that it was possible to change the light pattern used for printing the bilayer membrane to crosslink different areas in order to create complex structures to cover different bone defect sites, such as the femoral head and condyle (Figure 8Aii). Additionally, they applied an external heat source to reversibly change the topography of the shape-memory layer (microstructure switch) from flat to micropillar (Figure 8Aiii). Compared to the micropillar surface, the flat surface promoted higher cell density. However, after the proliferation stage, expression of osteogenic differential markers, i.e., alkaline phosphatase, type I collagen (Col-1), runt-related transcription factor 2 (RUNX2), osteocalcin (OCN), and mineralization suggested that the micropillar surface was a better choice for the differentiation stage. Transplanting this scaffold into a mouse bone defect model showed that compared to a scaffold with static flat or micropillar surface, the dynamic scaffold led to better walking performance after 4 weeks. This demonstrated that the dynamic change in the microstructure of the scaffold supported bone formation both *in vivo* as well as *in vitro*.

Enteroatmospheric fistula is an abnormal condition, often a complication of open abdomen, in which an opening in the gastrointestinal tract leads to leakage of its content to other tissues or organs. Previously proposed implants have struggled to fit into the bowel and have damaged intestinal mucosa. Using an extrusion-based printer, Qu et al. (2022) printed a layer of acrylamide-acrylic acid/cellulose nanocrystal (AAm-AAc/CNC) then exposed to UV for crosslinking. This layer was then stretched, and Fe^3+^ was incorporated to stabilize the acquired length through the coordination of Fe^3+^ and AAc. A second layer of AAm-AAc/CNC was then printed and polymerized using UV light. Then, the hydrogel was transferred to sodium lactate solution and irradiated with UV which led to de-coordination of the first layer and enabling the shape change. The bending degree could be controlled by the length of the first layer in the pre-stretched mode so that the scaffold could fit in the desired position. In the *in vivo* study, nine New Zealand white rabbits were divided into three groups: a control group (no transplantation), transplanted with thermoplastic polyurethane stents (TPU), and transplanted with as-prepared hydrogels (Figure 8B). To demonstrate *in vivo* performance, fistulas were first generated surgically through a terminal ileostomy. Animals in the experimental conditions received the respective stents or hydrogels to plug the fistula. Their results showed that after 36 h, the leakage of intestinal juice was 206 mL when thermoplastic polyurethane stents were used while it was reduced to 63 mL by the use of the bilayer hydrogels.

Another area that could benefit from shape-morphing scaffold is cardiac tissue engineering. A myocardial infarction could irreversibly damage myocardium and possibly cause heart failure. The integration of scaffolds into the heart is challenging due to the varying curvature across the tissue. To overcome this challenge, Wang et al. (2021) printed 4D myocardial tissues with adjustable curvature (Figure 8C). To create the scaffold, they fabricated a mold with microgroove arrays from PEGDA using digital light processing-based printing. Then, an 15% graphene-doped ink with a 10:3:4 molar ratio of bisphenol A diglycidyl ether, poly(propylene glycol) bis(2-aminopropyl) ether and decylamine was extruded onto the mold. Due to the presence of graphene in the ink, it was possible to spatiotemporally control the shape-changing property of the scaffold through near-infrared (NIR) exposure; as the straight sheets were irradiated with NIR light, they recovered their permanent U shape. To have the cell ratio of the heart, 4:2:1 ratio of human-induced pluripotent stem cell-derived cardiomyocytes (hiPSC-CMs), human ECs, and hMSCs were seeded on a flat scaffold. After an initial period of 7 days, shape change was actuated by NIR light exposure. Immunostaining showed evidence of myocardial protein, enhanced sarcomere density, and myocardial maturation. Additionally, the presence of microgrooves led to increased alignment of the cells and eventually synchronized beating.

### 4.3 Biosensors

Medical sensors have also been improved by the use of shape-changing materials. Current sensor models oftentimes provide less-than-optimal signal-to-noise ratios and conformability to intricate or mobile parts of the human body. Kirigami-inspired designs for 3D composites will revolutionize medical sensors by introducing characteristics like deformability, stretchability, conformability, and self-assembly (Brooks et al., 2022). These characteristics allow sensors to better adapt to body contours and movements while improving sample collection and signal-to-noise ratios.

#### 4.3.1 Biomimetic sensors

The existing array of biomimetic sensors, such as wearable health gadgets (e.g., smart watches fitness monitors) and dermal patches, often possess a rigid nature, failing to seamlessly conform to the skin’s contours. This leads to discomfort during extended use and susceptibility to extraneous signal interference (Hua et al., 2022). Engineered to imitate or reproduce functions or traits found within the human body, biomimetic sensors leverage biological concepts to amplify their sensing prowess. Developments utilizing shape-transforming characteristics have revolutionized biomimetic sensors, enhancing conformability and sensitivity.

**FIGURE 9:**
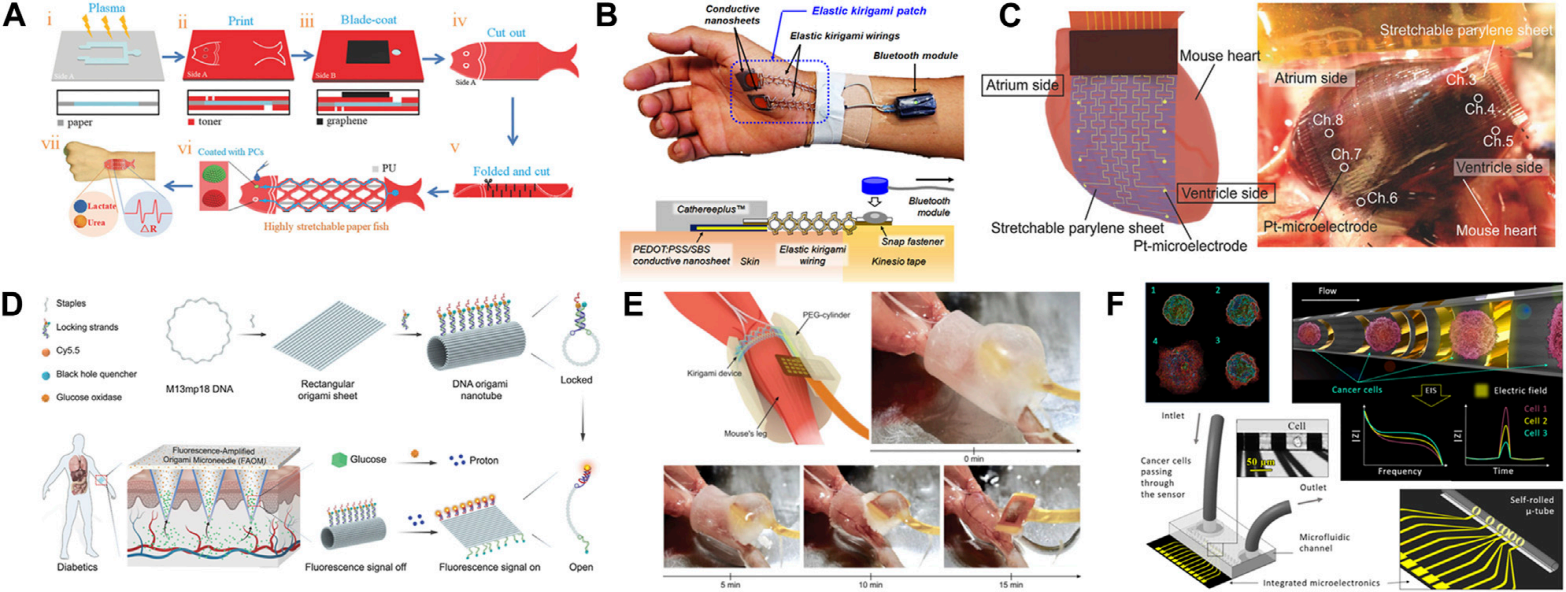
Shape-transformation in sensor applications. **(A)** Fabrication process of a wearable paper sweat sensor based on origami and kirigami enabled a shape change to detect lactic acid and urea (Gao et al., 2018). Reprinted with permission from (Gao et al., 2018) © 2018 WILEY-VCH Verlag GmbH & Co. KGaA, Weinheim **(B)** A kirigami-derived stretchable sensor incorporating conductive polymer nanosheet-based bioelectrodes for surface electromyography (EMG) (Yamagishi et al., 2019). Reprinted with permission from (Yamagishi et al., 2019) © 2019 The Authors. **(C)** A strategically cut parylene-C film could be bent out of plane to conform to the shape of the heart and record electrocardiograms (Morikawa et al., 2018). Modified with permission from (Morikawa et al., 2018) © 2017 WILEY-VCH Verlag GmbH & Co. KGaA, Weinheim. **(D)** Fabrication strategy (top) and working principle (bottom) of a DNA origami sensor that could be implanted under the skin to perform sensing using glucose oxidase incorporated within a microneedle (Li et al., 2023). Reprinted with permission from (X. Li et al., 2023) © 2023 Wiley-VCH GmbH. **(E)** Visual conformation of a kirigami donut EMG sensor to the leg of a mouse during *in vivo* testing (Morikawa et al., 2019). Modified with permission from (Morikawa et al., 2019) © 2019 WILEY-VCH Verlag GmbH & Co. KGaA, Weinheim. **(F)** A rolling shape change enabled the fabrication of a cell counting and viability sensing device integrated into microfluidic channel (Ghosh et al., 2022). Reprinted with permission from (Ghosh et al., 2022) © 2022 Walter de Gruyter GmbH, Berlin/Boston.

Humidity-sensing skin patches have been enhanced by the application of a bioinspired kirigami pattern resembling fish scales within a larger fish-shaped paper substrate (Gao et al., 2018). In contrast to prevailing rigid and non-conformable technologies, this fish-scale sensor exhibited stretchability and ventilation capabilities, concurrently facilitating sweat collection and diagnostic functionalities. The fabrication of the kirigami sensor involved subjecting regular office paper to plasma treatment to generate hydrophilic pathways, subsequently coated with a graphene ink via blade-coating (Figure 9A). A fish scale motif was intricately fashioned through cutting, folding, and symmetrical slitting with scissors. Fluorescence-enhancing silica photonic crystals were strategically placed on the eye location on the fish to enable heightened sensing of sweat components such as lactic acid and urea. Polyurethane strips were affixed to the fish-scale head and tail segments, thereby endowing the resultant paper-based sensor with a blend of elevated conductivity and stretchability. Notably, the adaptability across diverse patients is achieved through the sensor’s tunability, allowing for varied scale patterns with distinct stretch lengths. Furthermore, potential avenues for refining this fish-scale kirigami sensor encompass advancements like chip miniaturization and standardized machining techniques. Although the primary focus of this fish-scale sensor pertains to sweat level detection, the versatile kirigami technology demonstrated holds promise for integration into other wearable medical patches.

Wearable devices can also be used for surface electromyography (sEMG), a technique aimed at analyzing human movements and patterns. Presently, sEMG recording apparatuses consist of inflexible and cumbersome electrodes, coupled with connecting wires, which hinder the wearer’s natural movements and the accurate capture of sEMG signals during vigorous physical activities (Brooks et al., 2022). This issue becomes particularly pronounced when dealing with flexible body regions such as the palms, where proper conformity for precise sEMG recording proves to be challenging. In a study by Yamagishi et al. (2019), a solution to the mechanical incongruity between human skin and electrical sensors was achieved through the development of a skin-contact patch (Figure 9B). This patch integrates kirigami-derived stretchable wiring and conductive polymer nanosheet-based bioelectrodes possessing exceptional conformability. The approach uses a kirigami-inspired wiring configuration and a mechanical gradient structure along with nanosheet-based pliable bioelectrodes to create a conventional wearable unit. A shape transition in the form of stretching mitigates the mechanical strain exerted on the skin-contact bioelectrodes. This measurement system has demonstrated its utility in the context of baseball pitchers, capturing sEMG signals emanating from the abductor pollicis brevis muscle (APBM) during baseball pitches. This facilitated a comparative analysis of APBM activity across different pitch types, such as the fastball and curveball. Notably, it was discerned that electrode attachment to the palm region was susceptible to strain due to the wide wrist flexion angle (∼40°) accompanied by a rapid angular velocity of ∼5000°/s during a pitch. To surmount this challenge, a kirigami-based patch was devised employing 2D materials like graphene and carbon nanotube-infused nanocomposites. These materials, though inherently rigid and unstretchable in their natural state, were engineered to achieve stretchability through 3D deformations. Empirical testing validated the durability of this kirigami skin patch, exhibiting no noticeable damage even after subjection to five consecutive cycles of stretching and contracting, with a tensile strain reaching up to 200%. When juxtaposed with previous rigid skin patches, this sEMG measurement sensor allows increased options for exploring motion dynamics within flexibly muscle regions such as the palm. This technology harbors immense potential for gaining deeper insights into muscular activity spanning a diverse spectrum of athletic performances and medical muscular disorders aimed at comprehending intricate muscles including the feet’s soles, toes, fingers, and facial muscles.

#### 4.3.2 Implantable sensors

Intricate parts of the body, like the brain, heart, circulatory system, and muscular system have long been studied for accurate and comfortable monitoring. Current implantable sensors, which are designed to minimize invasiveness and monitor treatment or patient condition, often lack throughput, accuracy, and medical personalization (Brooks et al., 2022). Kirigami-inspired implantable sensors aim to improve these characteristics.

Morikawa et al. (2018) developed an ultra-stretchable bioprobe film device, consisting of multiple layers of inflexible materials, to mimic the intricate shapes of organs like the heart and brain (Figure 9C). Employing kirigami techniques, the bioprobe film is expanded through a pattern of strategically placed cuts, allowing the parylene-C film to stretch using an out-of-plane bending mechanism around each cut. Unlike previous technologies that relied on in-plane bending, such as pneumatic soft actuators, this out-of-plane bending technique achieves a higher strain at the same level of strain-force. During the cardiac cycle, the film undergoes significant stretching in sync with the heart’s large and rapid volume and surface area changes, occurring at a rate of approximately 2.5 cycles per second. In terms of sensing capabilities, they recorded epicardial electrocardiogram (ECG) signals from a beating mouse heart using an array of 10-channel planar Pt electrodes. For this purpose, a C57Bl mouse was anesthetized and after a thoracotomy, the heart was wrapped with the film device. A thorough investigation and analysis indicated the kirigami bioprobe could consistently record ECG signals from the beating heart with stability. Furthermore, the kirigami-based bioprobe showcased advantageous potential for conformably wrapping around brain tissue. In situations involving brain tissue growth, development, and pathological changes leading to deformations exceeding 300%, conventional stretchable devices made from elastic materials fall short in their ability to adequately accommodate the brain’s deformations. The proposed kirigami bioprobe, boasting a stretchability of over 1000%, substantially reduced the force required to stretch the film device. Due to this kirigami-based mechanism, this proposed sensor forms around individual hearts and brains with minimal invasiveness. This kirigami bioprobe film also displays promising results and techniques that can be applied to studies surrounding other intricate biological morphology such as the eyes and ears.

In another application in the circulatory system, a recent investigation introduced a fluorescence-amplified origami microneedle device to quantitatively monitor blood glucose levels (Li et al., 2023). While current portable glucometers (fingertip pricking sampling) and hospital blood biochemical analyzers (venous blood sampling) are effective in determining insulin administration details, their invasive nature can lead to tissue damage and wound infections. In recent years, the microneedle array patch has emerged as a cost-effective, painless, and minimally invasive alternative for transdermal insulin delivery. Despite the revolutionary success of the closed-loop insulin system with the microneedle patch, there remains a crucial need to monitor disease recovery or progression in real-time. This study addressed the challenge by constructing 2D rectangular DNA-origami sheet structures and designing multiple proton-driven DNA pairs with fluorophore/quencher components to shape DNA nanosheets into tubular structures (Figure 9D) (Li et al., 2023). The resulting fluorescence-amplified origami microneedle (FAOM) device incorporated glucose oxidase molecules and fluorescence pairs-containing DNA-origami nanotubes into its inner network structure. Upon insertion into the skin, the FAOM device efficiently collected glucose through needle-array structures and converted the input into proton signals through oxidase catalysis. Clinical blind measurements indicated that the FAOM device demonstrated a high level of accuracy (98.70% ± 4.77% standard deviation) compared to commercial blood biochemical analyzer.

Like previously mentioned complex biological components, devices used with the muscular system, such as electromyography (EMG) sensors, struggle with adhesion and precision. The current technology employed in EMG devices aims to achieve a level of stretchability and flexibility comparable to that of the intended muscular tissues with which they are meant to interface. However, traditional EMG electrodes made from elastomers possess a Young’s modulus approximately twenty times higher than that of muscles tissue. This inconsistency results in tissue surface displacement and inaccurate recordings of EMG signals. Addressing this issue, Morikawa et al. (2019) developed a novel bioprobe inspired by kirigami, taking the form of a donut shape (Figure 9E). This design mitigates device displacement and inaccurate signal readings. The process involves fabricating a two-dimensional kirigami film through photolithography, which is then transformed into a 3D cylindrical structure. However, a potential flaw in the design stems from the sharp edges of the tilted beams formed during the 3D transformation. While these edges have been demonstrated not to induce stress on tissues in various mouse trials, it is conceivable that when scaled up for human use, these sharp edges could lead to tissue stress. Furthermore, the bioprobe incorporates an array of microelectrodes seamlessly integrated into the 3D kirigami device, thanks to the two-dimensional microfabrication process. Research findings indicate that the donut-shaped kirigami device possesses an effective modulus of 76 kPa, which falls within the same range as the Young’s modulus of resting muscle tissues (5–40 kPa). By adjusting the dimensions of each beam in the design and the materials used, the effective modulus of the kirigami device can be further decreased to better suit small muscles (e.g., 1.5 kPa for the brain). When compared to the currently used elastomer-based stretchable devices, the kirigami design induces lower stress on tissues caused by the device (730 kPa for PDMS-based devices). For the *in vivo* study, a C57LB mouse was anesthetized, the skin and hamstring muscle of the right limb were removed, and the device was placed on the lower limb. In practical tests on mice, the kirigami donut-shaped bioprobe exhibited a displacement of less than 0.1mm, signifying a noteworthy enhancement over prior technologies. These collective attributes result in a stable and precise recording of EMG signals over prolonged periods, allowing for continuous and chronic applications while accommodating natural muscle deformation. Beyond this, the proposed sensor has the potential for adaptation and deployment across various parts of the body, facilitating prolonged monitoring not only for skeletal muscles but also for smooth muscles and other organs.

#### 4.3.3 *In vitro* sensors

Aside from the above-mentioned *in vivo* instruments, *in vitro* sensors have been developed inspired by kirigami for use with isolated materials or biological samples. Microfluidic networks may be used as sensors in the context of the micro total analysis system (µ-TAS), lab-on-a-chip (LOC), electrical impedance spectroscopy (EIS), and 3D tubular microelectrode systems. These label-free microfluidic networks display high sensitivity while acquiring results by separating and detecting various in-flow samples at multiple frequencies. Biosensors within these microfluidic systems allow for increased sensitivity due to close interaction of analytes and cells to system transducers through low volume of reagents and sample consumption as well as strategic design in the laminar regime. These methods provide precise ways to convert biological samples’ responses into readable physical signals (Egunov et al., 2021; Ghosh et al., 2022).

Ghosh *et al.* developed a self-assembled sensor-in-a-tube showing a significant advancement in microfluidic network cell counting, cell sorting, and in-flow detection (Ghosh et al., 2022). The proposed sensor-in-a-tube builds upon current microfluidic networks using a kirigami-inspired approach. With cervical cancer in mind, the sensor-in-a-tube was designed to monitor single cervical cancer cells’ (HeLa cells) viability and impact of a model drug on the cells with the intent to revolutionize rapid drug screening and personalized medicine applications (Figure 9F). During the investigation, different concentrations of Camptothecin were introduced to the single HeLa cells, resulting in various cell stage deaths reported by the sensor-in-a-tube. Based on this information, the sensor-in-a-tube provides an innovative and improved platform to further refine drug therapies and develop new drugs. Considering the experiment can be altered to study various types of cells throughout different diseases and personal cases, therapy and drug development can significantly enhance medicine.

## 5 Conclusion

Shape-changing structures provide the advantages of straightforward fabrication and patterning in a lower dimension followed by a transformation into a higher dimension for improved functionality. In the process of generating such structures, both well-established methods, i.e., lithography, and new additions to the fabrication toolbox, i.e., bioprinting, enable the formation and subsequent conversion of diverse materials of interest into functional structures. The ability to enact a change in the underlying structure, encoded through materials properties such as stress or relative hydrophobicity/hydrophilicity, allows for a transformation into the third dimension in space along the fourth dimension of time. We have summarized common structures constructed through rolling, gripping, and folding transformation. These and other transformations have produced solutions to challenges in different biomedical applications, mimicking biological structures and surfaces, enhancing the signal-to-noise ratio of sensors, and facilitating minimally invasive procedures.

As discussed throughout, the ultimate application can inform different design decisions and selection of techniques and material. Length scale and resolution of structure, biocompatibility, optical transparency, time scale of transformation, and reversibility of the actuation are all crucial considerations, and the ability to maintain advantageous properties in multiple of these areas while maximizing throughput an area for continued development. While the aforementioned design aspects have been studied extensively, other concerns remain under-investigated in designing for biological and biomedical applications. While gas permeability, particularly oxygen permeability, is a crucial factor for cell viability and function, it is often uncharacterized beyond brief mention in cell viability. Additionally, in some cases, such as culturing cells with high metabolic activity, recapitulating organs with different levels of oxygen in different regions such as the liver, and modeling tumor microenvironment with hypoxic regions, having more control over oxygen permeation is an important design consideration. The interaction of shape-changing materials and structures with the immune system is another factor that should receive more attention as this field matures, especially when the ultimate goal of some sensors and tissue scaffolds is to be implanted in the human body. Accordingly, aside from their functionality, possible acute or chronic inflammation should be thoroughly evaluated for structures designed with the intention of interfacing with the body. Additionally, incorporating immunomodulatory materials in these structures might be a way to enhance their *in vivo* compatibility. The on-going introduction of new materials, investigation of new stimuli, and improvements in fabrication methods suggest a bright future for shape-transforming materials as well as new avenues for their incorporation into biomedicine.

## Figures and Tables

**Table:** 

**APBM**	abd+A2:B43uctor pollicis brevis muscle
**ADSC**	adipose-derived stem cell
**Calcein-AM**	calcein-acetoxymethyl ester
**CMC**	carboxymethyl cellulose
**CMCS**	carboxymethyl chitosan
**CS**	chitosan
**CAD**	computer-aided design
**DTPA**	diethylenetriaminepentaacetic acid
**EIC**	electrical impedance spectroscopy
**EMG**	electromyography
**E-beam**	electron beam
**EC**	Endothelial cell
**ECG**	epicardial electrocardiogram
**EDTA**	ethylene-diaminetetraacetic acid
**ECM**	extracellular matrix
**FAOM**	fluorescence-amplified origami microneedle
**FDM**	Fused Deposition Modeling
**Ge**	germanium
**hiPSC-CMs**	human-induced pluripotent stem cell-derived cardiomyocytes
**IGZO**	indium gallium zinc oxide
**LOC**	lab-on-a-chip
**MAPLE DW**	matrix-assisted pulsed-laser evaporation direct-write
**MSC**	mesenchymal stem cell
**µ-TAS**	micro total analysis system
**HEAA**	N-(2Hydroxyethyl)acrylamide
**NIR**	near-infrared
**PLA**	polylactic acid
**OCN**	osteocalcin
**PHEMA**	poly(2-hydroxyethyl methacrylate)
**P(AAm-BAC)**	poly(acrylamide-N,N′-bis(acyloyl)cystamine)
**parylene-C**	poly(chloro-p-xylylene)
**PEG**	poly(ethylene glycol)
**PEGDA**	poly(ethylene glycol) diacrylate
**PEMA**	poly(ethylene-alt-maleic anhydride)
**PLA**	poly(lactic acid)
**PLGA**	poly(lactic-co-glycolic acid)
**PIPAAm**	poly(N-isopropyl acrylamide)
**pNI-PAM-AAc**	poly(N-isopropylacrylamide-co-acrylic acid)
**P(OEGMADSDMA)**	poly(oligoethylene glycol methyl ether methacrylate-bis(2-methacryloyl)oxyethyl disulfide)
**PSMa**	poly(styrene-co-maleic anhydride)
**PVA**	poly(vinyl alcohol)
**PCL**	polycaprolactone
**PDMS**	polydimethylsiloxane
**PPF**	polypropylene fumarate
**PAB**	pressure-assisted bioprinting
**RGO**	reduced graphene oxide
**RUNX2**	runt-related transcription factor 2
**SIRM**	stress-induced rolling membrane
**sEMG**	surface electromyography
**Col-1**	type 1 collagen
